# Targeting CDKs with Roscovitine Increases Sensitivity to DNA Damaging Drugs of Human Osteosarcoma Cells

**DOI:** 10.1371/journal.pone.0166233

**Published:** 2016-11-29

**Authors:** Serena Vella, Elisa Tavanti, Claudia Maria Hattinger, Marilù Fanelli, Rogier Versteeg, Jan Koster, Piero Picci, Massimo Serra

**Affiliations:** 1 Laboratory of Experimental Oncology, Orthopaedic Rizzoli Institute, Italy; 2 Department of Human Genetics, Academic Medical Center, University of Amsterdam, The Netherlands; 3 Department of Oncogenomics, Academic Medical Center, University of Amsterdam, The Netherlands; Universite de Nantes, FRANCE

## Abstract

Cyclin-dependent kinase 2 (CDK2) has been reported to be essential for cell proliferation in several human tumours and it has been suggested as an appropriate target to be considered in order to enhance the efficacy of treatment regimens based on the use of DNA damaging drugs. We evaluated the clinical impact of CDK2 overexpression on a series of 21 high-grade osteosarcoma (OS) samples profiled by using cDNA microarrays. We also assessed the in vitro efficacy of the CDKs inhibitor roscovitine in a panel of drug-sensitive and drug-resistant human OS cell lines. OS tumour samples showed an inherent overexpression of CDK2, and high expression levels at diagnosis of this kinase appeared to negatively impact on clinical outcome. CDK2 expression also proved to be relevant for *in vitro* OS cells growth. These findings indicated CDK2 as a promising candidate therapeutic marker for OS and therefore we assessed the efficacy of the CDKs-inhibitor roscovitine in both drug-sensitive and -resistant OS cell lines. All cell lines resulted to be responsive to roscovitine, which was also able to increase the activity of cisplatin and doxorubicin, the two most active DNA damaging drugs used in OS chemotherapy. Our results indicated that combined treatment with conventional OS chemotherapeutic drugs and roscovitine may represent a new candidate intervention approach, which may be considered to enhance tumour cell sensitivity to DNA damaging drugs.

## Introduction

Osteosarcoma (OS), the most common malignant tumour of bone, is usually treated with neoadjuvant chemotherapy protocols based on cisplatin (CDDP), doxorubicin (DX), methotrexate (MTX) and ifosfamide [1–3]. The fact that, despite this multidrug aggressive treatment, 35–40% of OS patients recur and experience an unfavourable outcome, claims for new treatments which may improve the presently achievable clinical results.

Deregulation of cell cycle control mechanisms and aberrant activities of cell cycle-related kinases have been associated with neoplastic evolution and progression of several human cancers, including OS [4–10]. Key regulators of the transition along cell cycle phases are the cyclin-dependent kinases (CDKs), a family of serine/threonine kinases that form heterodimeric complexes with cyclins and operate in distinct phases of the cell cycle playing a key role also in tumour cells proliferation [4, 5, 10, 11].

Regulation of CDKs activity occurs at multiple levels, and human cancer cells frequently present deregulated CDKs activities, which allows them to escape the normal cell cycle regulation machinery [4, 5, 10]. In particular, CDK2 has proved to be deregulated in various malignancies, thus appearing as a relevant factor for the uncontrolled proliferation of tumour cells [5, 6, 10–13].

CDKs are essential not only for cell cycle regulation and cell division, but also for cellular response to DNA damaging agents, with important consequences for chemotherapy response [14–18]. The increased activity of DNA damage repair mechanisms is one of the most relevant factor responsible for resistance to several of these drugs, which also include agents that are commonly used for OS chemotherapy as CDDP, ifosfamide and DX [19, 20]. These genotoxic agents produce different DNA alterations, which are sensed by signaling pathways that ultimately lead to CDKs inhibition and cell cycle arrest. Therefore, interfering with this system may improve the efficacy of DNA damaging drugs and indicate innovative therapeutic approaches.

For all these reasons CDKs have been considered as attractive targets for cancer therapy and several CDK inhibitors have been developed and are now available for clinical use [4, 5, 21, 22]. One of the most studied of these inhibitors is roscovitine (marketed as seliciclib or CYC202; Cyclacell Pharmaceuticals Inc, Berkeley Heights, NJ), a 2,6,9-tri-substituted purine analogue of olomoucine that competes with ATP for its binding site on CDK2 and other CDKs [4–6, 10, 23–25]. Seliciclib has been tested in Phase I and II clinical trials for haematologic and solid tumours, showing some promising results and indicating that combination therapies with conventional chemotherapeutic drugs were more effective than monotherapy regimens [2, 4–6, 21, 25–27].

Data about CDK2 impact and relevance in OS, as well as information on CDKs inhibitors acitivity in this tumour, are still very scarce. In the present study, we have assessed the biologic relevance of CDK2 expression in OS cells with the aim to define whether this kinase may be considered as a new candidate target for therapeutic interventions based on the use of roscovitine. The efficacy of roscovitine has then been tested on a panel of drug sensitive and resistant human OS cell lines, which were treated with either roscovitine alone or in combination with the drugs used in conventional OS chemotherapy.

## Materials and Methods

### Drugs

CDDP, DX, and MTX were purchased, respectively, from Teva Italia (Milan, IT), Wyeth Lederle (Latina, IT) and Sandoz (Varese, IT). Roscovitine was purchased by Santa Cruz Biotechnology (Dallas, TX). Stock solutions of CDDP (500 μg/ml) and MTX (25 mg/ml) were stored at 4°C. Stock solution aliquots of DX (2 mg/ml) were stored at -20°C. Roscovitine was dissolved in DMSO at 10 mM concentration and stock solution aliquots were stored at -20°C. For all drugs, working concentrations were prepared by diluting stock solutions in culture medium immediately before use.

### Cell lines

The cell line panel used for this study included seven drug-sensitive (U-2OS, Saos-2, IOR/OS9, IOR/OS10, IOR/OS14, IOR/OS18, and SARG) and six drug-resistant human OS cell lines. U-2OS, Saos-2, HOS (CRL-1543) and MG-63 cell lines were obtained from the American Type Culture Collection (ATCC, Rockville, MD). IOR/OS9, IOR/OS10, IOR/OS14, IOR/OS18, and SARG human OS cell lines were established from bioptic clinical specimens obtained from OS patients at the Laboratory of Experimental Oncology of the Rizzoli Orthopaedic Institute [28]. Variants resistant to DX (U-2OS/DX580 and Saos-2/DX580), MTX (U-2OS/MTX300; Saos-2/MTX300), and CDDP (U-2OS/CDDP4μg and Saos-2/CDDP6μg) were established by exposing the drug-sensitive U-2OS and Saos-2 parental cell lines to stepwise increasing concentrations of each drug, as previously described [29–31]. DNA fingerprint analysis of 14 polymorphic short tandem repeat (STR) sequences was performed for all cell lines as previously described [32]. STR profiles of drug resistant variants were identical to those of their corresponding parental cell lines. All cell lines were cultured in Iscove's modified Dulbecco's medium (IMDM), supplemented with penicillin (20 U/ml), streptomycin (20 U/ml) (Invitrogen Ltd, Paisley, UK) and 10% heat-inactivated fetal bovine serum (FBS; Biowhittaker Europe, Cambrex-Verviers, BE), and maintained at 37°C in a humidified 5% CO_2_ atmosphere.

### Clinical samples

The series of clinical samples used in this study included 21 conventional OS (primary, high-grade tumours located in the extremities of patients younger than 40 years of age). Samples were obtained from surgical biopsies at diagnosis and patients were successively treated with neoadjuvant chemotherapy protocols based on DX, MTX, CDDP and ifosfamide. After the end of chemotherapy treatment, patients were continuously followed and clinical data updated. Adverse events were defined as recurrence of the tumor at any site or death during remission, and event-free survival was calculated from the date of initial diagnosis. Median follow-up was 91 months (range 63–218 months). Written informed consent for using their biologic material for research purposes was obtained from each patient entering the study. The study was approved by the institutional Ethics Committee.

### RNA isolation

RNA was isolated from both human OS cell lines and clinical samples. Before extraction, all clinical samples were histologically examined for tissue quality in order to isolate RNA only from representative specimens containing at least 90% tumour cells. RNA was extracted by using TRIzol reagent (Invitrogen Ltd) according to standard procedures. After isolation, RNA concentration and quality were evaluated by spectrophotometry using NanoDrop ND-1000 (NanoDrop Technologies, Wilmington, DE) and by electrophoresis on a 1.5% agarose gel. Fragmentation of cRNA, hybridization to hg-u133 plus 2.0 microarrays and scanning were performed as previously described by [33]. After normalization of expression data using the MAS5.0 algorithm, gene expression profiles were analysed and visualised with the freely available R2 web application (http://r2.amc.nl).

### CDK2 silencing by siRNA

Cells were seeded in 6-well plates in drug-free IMDM 10% FBS without antibiotics. After 24 h, medium was replaced with FBS- and antibiotic-free IMDM supplemented with Lipofectamine 2000 (Invitrogen Ltd) and 25 nM Dharmacon ON-TARGET plus SMARTpool siRNA (Thermo Fisher Scientific, Waltham, MA) specific for CDK2 (J-003236-00, Human CDK2) or scrambled SMARTpool siRNA (D-001810-02-20). Controls were cultured in the same media without siRNA. After 5 h, transfection medium was replaced with IMDM 10% FBS without antibiotics and cells were maintained in culture for additional 24–96 h. After evaluation of cell morphology, cells were harvested, counted with the trypan blue dye exclusion method, and processed for cell cycle analysis and for RNA and protein extraction.

### Quantitative reverse transcriptase-polymerase chain reaction (qRT-PCR)

For single gene expression analyses, 500 ng of total RNA were reverse transcribed using the High Capacity cDNA Archive Kit (Applied Biosystems, Foster City, CA) according to the manufacturer’s protocol. cDNAs were aliquoted and stored at -20°C until use. To quantify the fold-change in gene expression between silenced samples and controls, the TaqMan Gene Expression Assay CDK2 Cyclin-dependent kinase 2 (Assay ID: Hs00608082_m1; Applied Biosystem) was used on the ABI PRISM 7900 SDS instrument (Applied Biosystem). GAPDH (Assay ID: Hs99999905_m1; Applied Biosystem) was used as reference gene.

### Western blot

Cells were scraped, washed twice in cooled phosphate buffered saline solution (PBS) and then lysed in RIPA buffer or lysis buffer for phosphoproteins containing 50mM Tris HCl pH 7.4, 150 mM NaCl, 1% NP-40, 0.25% NaDesoxycholate, 1mM EGTA, 1mM NaF, and inhibitors (0.2 mM Na_3_VO_4_, 1mM phenylmethylsulfonyl fluoride, and 10 μg/mL aprotinin). Cell suspensions were shaked in ice for 30 minutes. Lysates were centrifuged at 13,000 rpm for 15 min at 4°C. Equal amounts of cell lysates were dissolved with SDS-PAGE and then transferred to PVDF membranes (Immobilon P-Transfer membrane, Millipore, Billerica, MA). Membranes were incubated in blocking solution consisting of 5% powered milk in TBST solution (Tris-Buffered Saline and Tween 20) at room temperature for 1 h and then with one of the following primary antibodies: 1:1000 anti-CDK2 mouse monoclonal antibody (catalog number #610146, BD Transduction Laboratories, San Diego, CA), 1:1000 Poly ADP-ribose polymerase-1 (rabbit polyclonal PARP-1, catalog number #9542) and 1:1000 Cleaved Caspase-3 (Asp175) (rabbit polyclonal, catalog number #9661) both purchased from Cell Signaling Technology (Danvers, MA). To verify the protein loading of each sample, the same membranes were immunostained with 1:50000 mouse anti-actin, clone C4, monoclonal antibody (catalog number #MAB1501, Merck Millipore, Darmstadt, DE). Protein bands were visualized by using an enhanced chemiluminescence detection system (Liteablot® Plus, Euroclone, Milan, IT or Immobilon Western, Millipore, Billerica, MA).

### In vitro drug efficacy

The *in vitro* sensitivity to roscovitine was estimated on the basis of drug dosage response curves, assessed by using the 3-(4,5-dimethylthiazol-2-yl)-2,5-dephenyltetrazolium bromide (MTT) assay kit (Roche Diagnostics GmbH, Mannheim, DE) or the CellTiter-Fluor Cell Viability Assay kit (Promega Corporation, Madison, WI) after 96 h of drug treatment. For each cell line, the IC50 value (drug concentration that induced 50% growth inhibition compared to untreated controls) was determined.

### Evaluation of drug-drug interactions

To evaluate *in vitro* interactions between roscovitine and conventional chemotherapeutic drugs, human OS cell lines were incubated with different regimens of two-drugs combinations. Cell lines were treated with combinations of increasing drug dosages defined by the ratio of the specific IC50 values obtained for each cell line. Drug interaction effects were evaluated after 96 h of combined treatment. In drug sequence experiments, cell lines were sequentially exposed for 24 h to increasing dosages of DX or CDDP and then to increasing dosages of roscovitine for additional 48 h. The type of interaction in terms of synergism, antagonism or additivity, was defined on the basis of the combination index (CI) of each two-drugs combination, which was calculated with the equation of Chou-Talalay by using the CalcuSyn software (Biosoft, Stapleford, UK). By following the range of CI values indicated in the CalcuSyn software manual, we classified the drug–drug interaction as synergistic when CI was lower than 0.90, as additive when 0.90≤CI≤1.10, or as antagonistic when CI was higher than 1.10.

### Cell cycle analysis

Assessment of drug-induced cell cycle perturbations was performed by seeding drug-sensitive and -resistant cell lines in IMDM 10% FBS. After 24 h, medium was changed with IMDM 10% FBS without (control) or with the IC50 concentration of DX (U-2OS: 0.01μM, Saos-2: 0.01μM, U-2OS/DX580: 7.21μM, Saos-2/DX580: 8.21μM) or CDDP (U-2OS: 2.5μM, Saos-2: 1.1μM, U-2OS/CDDP4μg: 26.9μM, Saos-2/CDDP6μg: 30.8μM) for 24 h and then to the IC50 concentrations of roscovitine for additional 48 h. Additional controls were also included by culturing cells in IMDM 10% FBS additioned with DMSO concentrations corresponding to those of drug-treated samples. At the end of drug exposure, cells were incubated with 10 mM bromodeoxyuridine (Sigma-Aldrich Co., St. Louis, MO) for 1 h in a humidified 5% CO_2_ athmosphere at 37°C, harvested, and fixed in 70% ethanol for 30 min. After DNA denaturation with 2N HCl, cells were processed for indirect immunofluorescence with the B44 anti-bromodeoxyuridine mouse monoclonal antibody (Becton Dickinson, San Jose, CA) diluted 1:8, followed by an anti-mouse FITC antibody (Sigma-Aldrich Co.) diluted 1:200. For the simultaneous determination of DNA content, cell nuclei were counterstained with 20 μg/ml propidium iodide (Sigma-Aldrich Co.). All samples were analysed by flow cytometry (FACSCalibur, Becton Dickinson).

### Alkaline comet assay

To estimate the extent of DNA damage on individual cells, the alkaline comet assay was chosen on the basis of its sensitivity and flexibility [34]. All cell lines were treated with their respective IC50 drug dosages. Time-course, setting experiments were performed to determine the time-points at which the treatment with DX and CDDP produced the higher tail moment, corresponding to extensive DNA damage. The influence of roscovitine on DNA repair activity was assessed after the treatment with DX or CDDP by incubating cells with the IC50 concentration of roscovitine for 1h and 3h.

The alkaline comet assay was conducted according to the procedure developed by [35], with a few modifications. After drug treatments, cells were harvested and counted with the Trypan Blue dye exclusion method. Cells were then mixed with a suspension of 1% low gelling temperature agarose (Sigma-Aldrich Co.) and spread on slides coated with ultrapure agarose (Invitrogen Ltd). After agarose solidification, slides were incubated in lysis solution (pH 13) for 1.5–2 h, immersed for 30 min in an electrophoresis buffer at 4°C, and subjected to electrophoresis. Samples were stained with 0.2 μg/ml propidium iodide in PBS and analysed with a fluorescence microscope (Nikon Eclipse 90i, Chiyoda-Tokyo, JP). For each experimental point at least 100 cells were imaged and comet tails (cells with residual DNA damages) were analysed and quantified with Casp-Comet Assay Software Project (1.2.2beta version). Since the relative length and intensity of DNA tails to heads is proportional to the amount of DNA damage present in each individual nucleus, the amount of DNA breaks was quantified by calculating the tail moment (TM), which is the product of the tail length and the fraction of total DNA in the tail.

### Statistics

Differences among means were analysed by Student’s t test. Kaplan-Meier and log-rank methods were used to draw and evaluate the significance of survival curves. The non parametric Mann-Whitney test was used to assess the significance of the difference in DNA-damage quantified on the basis of the evaluation of the TM. Significance was set at P ≤ 0.05.

## Results

### Analysis of CDK2 expression level

**Fig 1:**
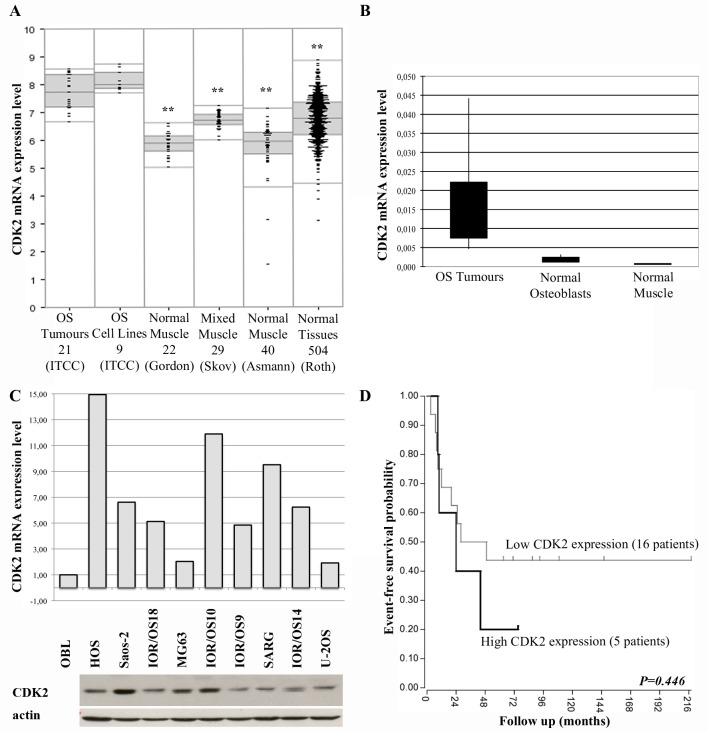
CDK2 gene expression analyses in human osteosarcoma. **A)** CDK2 expression in high-grade osteosarcoma tumour samples and cell lines in comparison with normal tissues. Numbers indicate the samples analysed in each group. Asterisks (**) indicate statistically significant differences with the osteosarcoma tumour samples *(P<0*.*01 by ANOVA test)*. The bottom and top of each box are, respectively, the first and third quartiles of gene expression level values. The band inside each box is the median (second quartile) of gene expression level values, whereas outliers (values below the first or above the third quartile) are plotted outside the box. The "OS Tumors 21 (ITCC)" data set was a series of 21 primary, high-grade osteosarcomas of the extremities, non-metastatic at diagnosis, arisen in patients younger than 40 years collected at the Orthopaedic Rizzoli Institute (IOR, Bologna, Italy) (GEO ID: GSE87437). The "OS Cell Lines 9 (ITCC)" data set included nine IOR human osteosarcoma cell lines. Both data sets were shared within the Innovative Therapies for Children with Cancer European Consortium (ITCC; http://www.itcc-consortium.org/). The GEO ID of the other data sets are: Normal Muscle 22 (Gordon) (GEO ID: GSE38718), Mixed Muscle (skeletal) 29 (Skov) (GEO ID: GSE6798), Mixed (skeletal) Muscle 40 (Asmann) (GEO ID: GSE9103), Normal Tissues 504 (Roth) (GEO ID: GSE7307). **B)** CDK2 expression level in high-grade osteosarcoma tumour samples in comparison with normal human normal osteoblasts and normal muscles assessed by qRT-PCR. **C)** Relative CDK2 gene expression level in the nine human osteosarcoma cell lines of the "OS Cell Lines 9 (ITCC)" data set, assessed by qRT-PCR and western blot. **D)** Event-free survival probability of the 21 high-grade osteosarcoma patients (GEO ID: GSE87437) stratified according to the CDK2 gene expression levels. The group of high expressors included patients with CDK2 expression levels at diagnosis equal or higher to the 75 percentile of the whole group. Curves were drawn by using the Kaplan-Meyer method and significance was calculated with the log-rank test.

Twenty-one cases of primary high-grade OS were profiled and data were analysed with the R2 bioinformatic tool (http://r2.amc.nl). Gene expression profiling analyses showed that CDK2 expression level was significantly higher in OS samples in comparison to human normal muscles and other normal tissues (Fig 1A). Comparison of CDK2 expression between OS clinical samples and human normal osteoblasts by qRT-PCR showed a remarkable higher kinase expression in tumours compared to osteoblasts (Fig 1B). CDK2 mRNA expression level in our series of human OS cell lines was overlapping with that of clinical samples (Fig 1A), and showed an inherent variability and an incomplete concordance with the corresponding protein level (Fig 1C).

Survival analyses showed that high CDK2 expression level at diagnosis was associated with a trend toward a worse outcome in terms of event-free survival probability (Fig 1D).

This body of information indicated that CDK2 is inherently overexpressed in OS cells and that its increased expression appears to negatively impact on clinical outcome.

### CDK2 silencing

**Fig 2:**
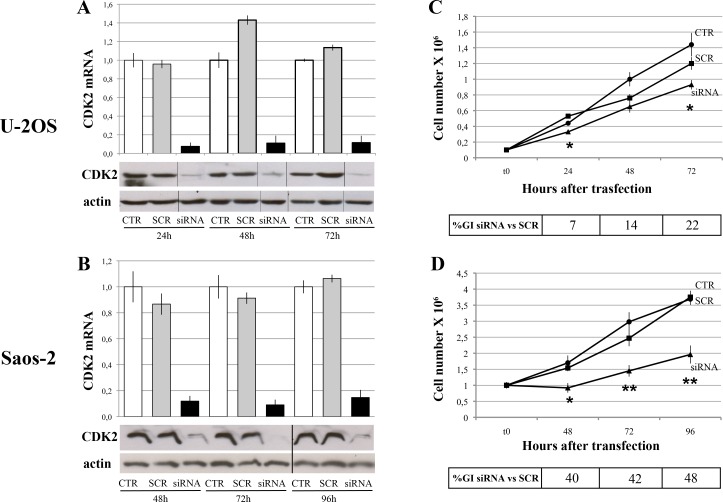
**CDK2 gene and protein knock-down after siRNA transfection** in U-2OS (A) and Saos-2 (B) human osteosarcoma cell lines. CDK2 mRNA and protein levels were assessed at different time points after the end of siRNA treatment (from 24 to 72 h for U-2OS; from 48 to 96 h for Saos-2). At the same time points, the extent of growth inhibition induced by CDK2 silencing was also estimated (C-D). Asterisks indicate statistically significant differences between SCR and siRNA treatments (** *P<0*.*01* and * 0.*01<P<0*.*05* by Student’s t test). Data refer to three different experiments and western blot images are representative of these determinations. Black lines show where the original gel (S1 Fig) was cropped to obtain the final image. Legend: CTR, control, not-treated cells; SCR, cells transfected with scrambled siRNA; siRNA, cells transfected with anti-CDK2 siRNA; h, hours after siRNA transfection; %GI, percentage of growth inhibition.

In order to determine whether CDK2 expression is relevant for OS cells growth, RNA interference approaches were used to knock-down this gene in U-2OS and Saos-2 OS cell lines. Treatment of these two cell lines with anti-CDK2 siRNA proved to significantly knock-down this kinase, both at mRNA and protein level (Fig 2A and 2B). The silencing specificity was confirmed by the fact that transfection with scrambled siRNA did not decrease the CDK2 expression compared to untreated control cells.

The effect of CDK2 silencing was estimated through the evaluation of cell growth and proliferation. CDK2 knock-down produced cell growth inhibition, which was more relevant in Saos-2 cells (Fig 2C and 2D). These findings may indicate that the higher CDK2 expression in Saos-2 compared to U-2OS cells (Fig 1C) is related to a higher dependancy of Saos-2 cell growth from this kinase, leading to an increased sensitivity of Saos-2 cells to CDK2 gene silencing.

These results indicated that CDK2 expression is functionally and biologically relevant for OS cells growth and, therefore, this kinase may be considered as an interesting candidate therapeutic marker to be targeted by specific inhibitor drugs.

### In vitro sensitivity to roscovitine

**Fig 3:**
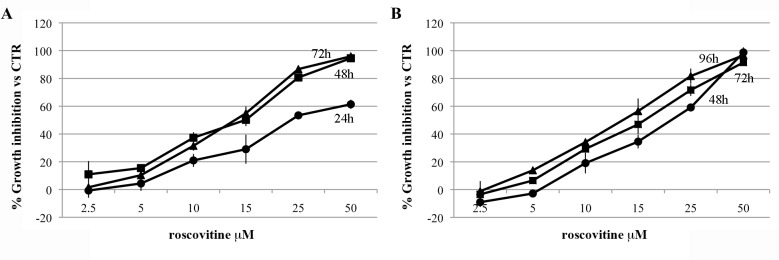
**Dose-response curves for roscovitine on U-2OS (A) and Saos-2 (B) osteosarcoma cell lines.** Cells were treated with increasing doses of roscovitine and growth inhibition was assesed at 24-, 48- and 72 h in U-2OS and 48-, 72- and 96 h in Saos-2. Data refer to three different experiments.

The in vitro activity of roscovitine was estimated on a panel of four drug-sensitive and six drug-resistant human OS cell lines (Table 1). Roscovitine proved to be active in all cell lines, which showed a clear dose- and time-dependent responsivity (Fig 3) and IC50 values in the low micromolar range (Table 1). No evidence of cross-resistance mechanisms was found, exhibiting all drug-resistant variants IC50 values similar or lower to those of their parental cells (Table 1). Only Saos-2/DX580 showed a lower CDK2 protein expression compared to the parental cells. Since no significant difference in CDK2 expression was found in all the other drug-resistant variants compared to their parental cell lines (S2 Fig), the fact that some drug-resistant variants exhibited lower IC50 values than the parental cells may indicate that their growth is more dependant from CDK2/CDKs activity than that of parental cells.

**Table 1:** *In vitro* sensitivity to roscovitine of drug-sensitive and drug-resistant human osteosarcoma cell lines.

Cell line	Mean IC_50_ value (μM)	SD
***Drug-sensitive***		
U-2OS	14.2	*5*.*3*
Saos-2	12.9	*2*.*3*
IOR/OS9	14.4	*2*.*7*
IOR/OS18	11.7	*3*.*4*
***Drug-resistant***		
U-2OS/DX580	7.0	*2*.*1*
Saos-2/DX580	17.0	*4*.*4*
U-2OS/MTX300	3.3	*0*.*6*
Saos-2/MTX300	5.5	*1*.*0*
U-2OS/CDDP4μg	12.1	*5*.*2*
Saos-2/CDDP6μg	14.3	*2*.*4*

IC50 values were calculated after 96 h of drug treatment. Data refer to the mean IC50 with the corresponding standard deviation (SD) of at least three different experiments.

Roscovitine was also used in combination with the conventional drugs that are most commonly used in OS chemotherapy, in order to verify whether these combined treatments may produce positive interactions. As shown in Table 2 and S3 Fig, the best interactions were obtained when roscovitine was sequentially administered after DX or CDDP. Association with CDDP produced mostly negative effects. Association with MTX proved to be almost invariably antagonistic, whereas mainly additive effects were observed when roscovitine was sequentially administered after this drug.

**Table 2:** Interaction of roscovitine with doxorubicin (DppX), methotrexate (MTX) and cisplatin (CDDP) in drug association and sequential exposure experiments.

Treatment schedule	U-2OS	Saos-2	U-2OS/DX580	Saos-2/DX580	U-2OS/MTX300	Saos-2/MTX300	U-2OS/CDDP4μg	Saos-2/CDDP6μg
***Drug association***								
Roscovitine + DX	SYN 0.42	SYN 0.66	SYN 0.32	ADD 1.06				
Roscovitine + MTX	Ant 1.63	Ant 1.50			Ant 1.24	Ant 1.80		
Roscovitine + CDDP	Ant 1.80	Ant 1.59					Ant 1.41	ADD 0.95
***Drug sequence***								
DX → Roscovitine	ADD 0.91	SYN 0.32	SYN 0.73	SYN 0.80				
MTX → Roscovitine	ADD 1.08	Ant 1.60			ADD 0.95	ADD 1.03		
CDDP → Roscovitine	SYN 0.44	SYN 0.57					SYN 0.61	ADD 1.06

Legend: SYN, synergistic; ADD, additive; ANT, antagonistic. Values indicate the combination index. Data refer to at least two different determinations.

### Cell cycle alterations and apoptosis induction of combined treatments

**Fig 4:**
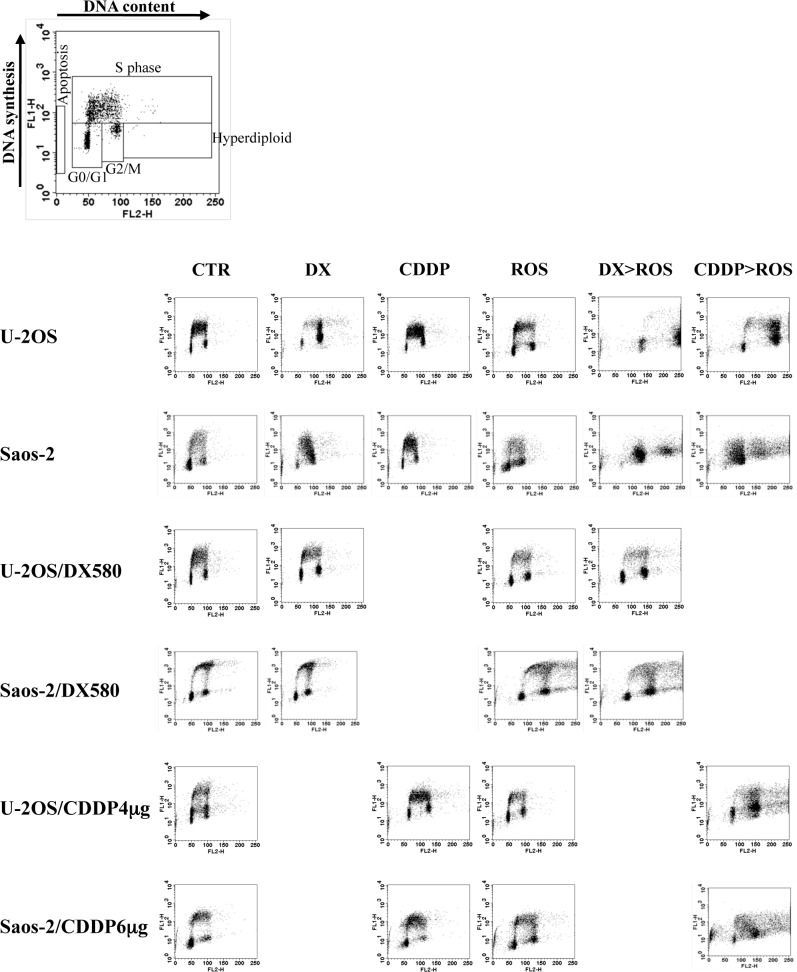
**Effects on cell cycle of doxorubicin (DX), cisplatin (CDDP) and roscovitine (ROS)**, used either alone or in combination, in the human osteosarcoma cell lines U-2OS and Saos-2 and their variants resistant to DX or CDDP. Cell cycle phase distribution was determined by flow cytometry after bromodeoxyuridine (BrdU) incorporation and propidium iodide counterstaining. The intensity of propidium iodide fluorescence (representative for the DNA content) was plotted on the X axis. The intensity of the incorporated BrdU fluorescence (representative for the DNA synthesis) was plotted on the Y axis. Legend: CTR, control cells cultured in drug-free medium; DX, CDDP, ROS, cells treated with their respective IC50 dosages of each drug for 24 h (DX and CDDP) or 48 h (ROS); DX>ROS and CDDP>ROS, cells sequentially treated with their respective IC50 dosages of DX or CDDP for 24 h followed by treatment with the IC50 concentration of ROS for 48 h.

To further explore the basis of the positive interactions obtained when roscovitine was sequentially administered after DX or CDDP, the effects on cell cycle and apoptosis of these combined treatments with equitoxic drug concentrations were evaluated in both parental cell lines and variants resistant to these two drugs (Fig 4 and S1 Table).

In the drug-sensitive, parental U-2OS and Saos-2 cell lines, DX treatment produced a remarkable decrease of the G0/G1 phase with a G2/M accumulation and, in Saos-2 cells, also a partial blockage in S phase. In both cell lines, CDDP produced blockage of cells in S phase. Treatment with roscovitine induced an increase in G0/G1 phase in U-2OS cells and a G2/M accumulation in Saos-2 cells, with a general alteration of cell cycle phases less remarkable than that produced by DX.

In DX-resistant variants, treatment with roscovitine induced an increase in G0/G1 phase in U-2OS/DX580, which was similar to that observed in parental cells, whereas in Saos-2/DX580 a G2/M accumulation with the appearence of a hyperploid population and a partial blockage in S phase was observed.

In CDDP-resistant variants, treatment with roscovitine produced an increase of G0/G1 phase in U-2OS/CDDP4μg and an accumulation in G2/M in Saos-2/CDDP6μg cells, which were simialr to those observed in parental cells.

Sequential exposure of parental cell lines to DX followed by roscovitine led to the appearence of an hyperdilpoid cell population, which most probably were not able to re-enter cell cycle, producing a concomitant decrease of S phase, whereas G2/M accumulation was observed only in Saos-2 cells. In the U-2OS/DX580 variant this combined treatment produced a decrease of S phase similar to that observed in U-2OS cells, with a concomitant G2/M increase but without the appearence of an hyperdiploid cell population. Saos-2/DX580 variant behaved similarly to its parental cell line, showing the presence of an hyperdilpoid cell population with a concomitant G2/M accumulation.

Sequential exposure of parental cell lines to CDDP followed by roscovitine increased cell hyperploidization, which is Saos-2 cells was also associated with a G2/M accumulation and a partial blockage in S phase. In the U-2OS/CDDP4μg variant, this combined treatment produced a hyperploidization as for U-2OS cells, which in these cells was also associated to a G2/M accumulation. In Saos-2/CDDP6μg cells, together with a hyperploidization and a partial blockage in S phase similar to those observed in parental cells, an increase of apoptotic cells was also oberved.

**Fig 5:**
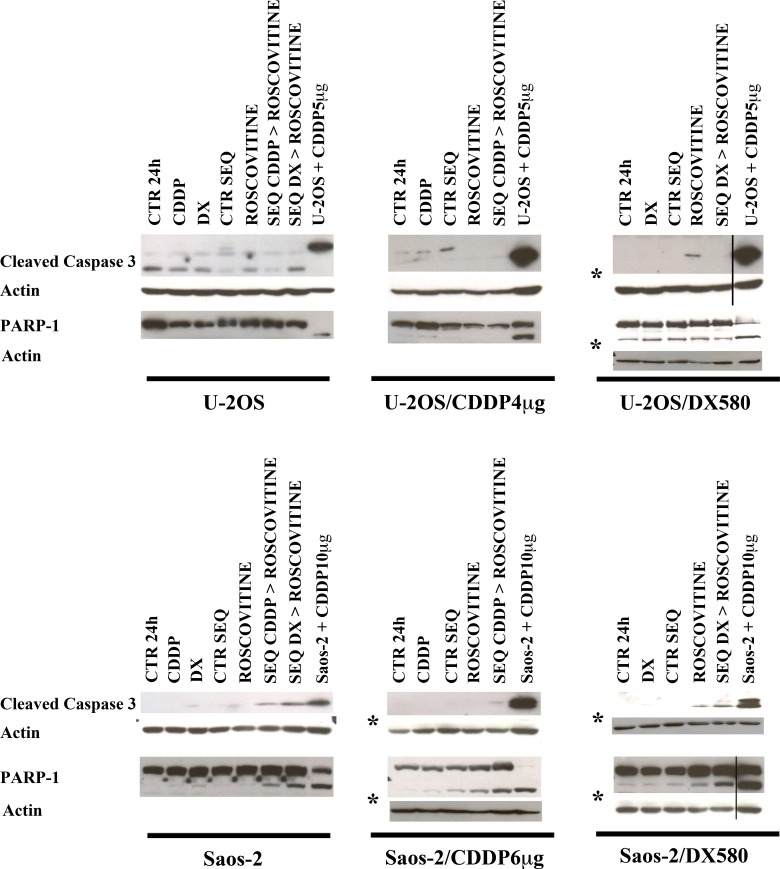
**Effects on apoptosis of sequential administration of doxorubicin (DX) or cisplatin (CDDP) followed by roscovitine (ROS)** assessed with western blot for cleaved caspase 3 and PARP-1. Positive controls (last lane) are represented by U-2OS and Saos-2 cell lines treated, respectively, with 5 μg/ml (16.7μM) or 10 μg/ml CDDP (33.3μM) for 48 h. Black lines indicate where the original gel was cropped (S4 Fig). Cleaved caspase 3 positive control of U-2OS/DX580 was the same of U-2OS/CDDP4μg, since all treatments of these cell lines were in the same gel. PARP-1 positive control in Saos-2/DX580 is cropped because it also was in the same gel, but far from the last treated sample. Asterisks (*) indicate different membranes of the same WB experiment. Images are representative of three different experiments. Legend: CTR 24h, control cells cultured in drug-free medium for 24 h; CTR SEQ, control cells cultured in drug-free medium for the total time of combined treatment (72 h). DX, CDDP, ROS, cells treated with their respective IC50 dosages of each single drug. SEQ DX>ROSCOVITINE, SEQ CDDP>ROSCOVITINE, cells treated with sequential administration of their respective IC50 dosages of DX or CDDP (for 24 h) followed by the IC50 concentration of ROS (for 48 h).

To further explore whether these drug treatments induced apoptosis through caspase 3 or PARP-1 pathways, cleaveage of caspase 3 and PARP-1 was assesed by western blot. As shown in Fig 5, apoptosis induction through caspase 3 and PARP-1 cleavage was detected only in Saos-2 cell lines, in which the sequential exposure schedules amplified this effect that was already evident after single drug treatments. No significant caspases 3 and PARP-1 cleavage was observed in U-2OS cell lines.

### Impact of roscovitine treatment on DNA repair

**Fig 6:**
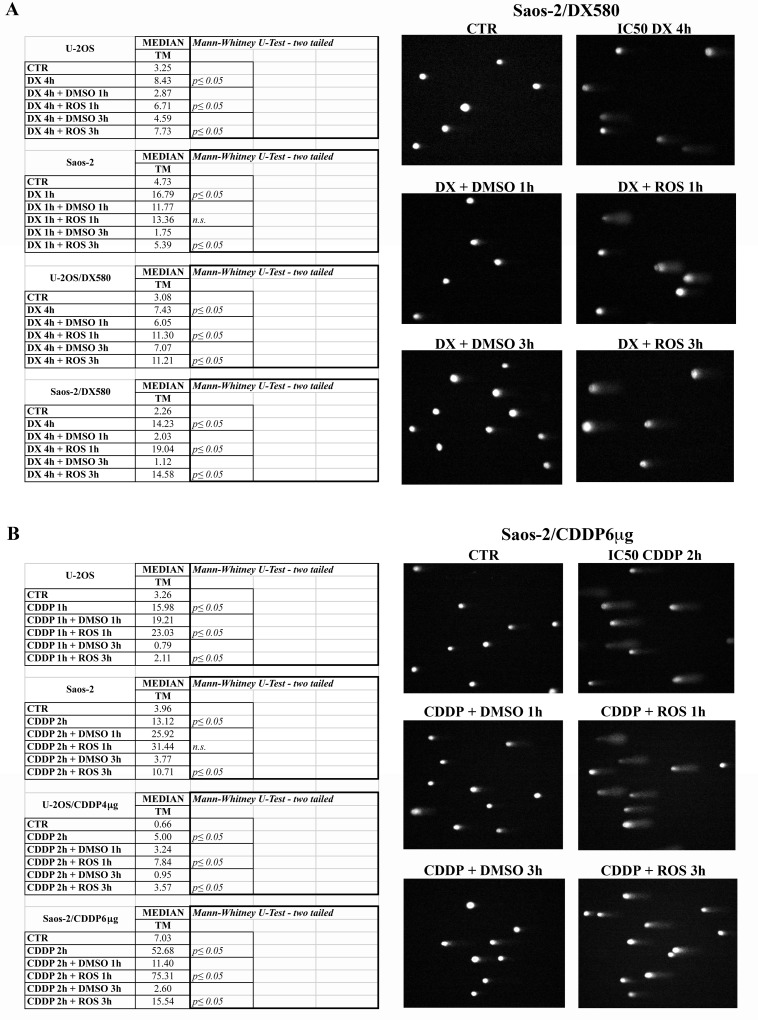
Impact of roscovitine treatment on DNA damage repair activity quantified by the alkaline comet assay. Drug-sensitive (U-2OS and Saos-2) and resistant (U-2OS/DX580, Saos-2/DX580, U-2OS/CDDP4g and Saos-2/CDDP6μg) human osteosarcoma cell lines were treated with their respective IC50 dosages of doxorubicin or cisplatin either alone or followed by administration of the IC50 dosage of roscovitine. The entity of DNA damage was quantified by calculating the tail moment (TM, the product of the tail length and the fraction of total DNA inside the tail) in 100 cells per sample by using the CaspLab computer software system. Data refer to three different experiments. Significance of differences between drug-treated cells and their corresponding untreated controls was calculated with the Mann-Whitney U-Test (left side of Fig 6A–B). Right side of Fig 6A–B shows representative images of Saos-2 drug resistant cell lines, which presented the most relevant retardation in DNA repair activity after roscovitine treatment. Legend: CTR, control cells cultured in drug-free and DMSO-free medium; DMSO, DMSO concentration corresponding to those of drug-treated samples; DX, doxorubicin; CDDP, cisplatin; ROS, roscovitine; h, hours of drug treatment; n.s., not significant.

Since the catalytic activities of CDKs play a critical role in drug-induced DNA damage response [36], we investigated whether the roscovitine-mediated CDK2 inhibition could interfere with this process also in OS cells. The entity of drug-induced DNA damage was assessed by using the alkaline comet assay [34] on parental cell lines and resistant variants after treatment with DX or CDDP administered either alone or followed by roscovitine. As shown in Fig 6A and 6B, sequential treatment with roscovitine produced a retardation in DNA repair of damages induced either by DX or CDDP in both drug-sensitive and resistant cell lines, while roscovitine alone was not able to induce DNA damage. Accordingly, the TM significantly increased in all samples which were sequentially treated with either DX or CDDP followed by roscovitine compared to those treated with DX or CDDP alone.

Overall, these results provided the evidence that roscovitine treatment delays the normal activation of DNA repair systems, leading to an accumlation of DNA double strand breaks and, consequently, to an increase of the activity of DNA damaging drugs, as DX and CDDP.

## Discussion

Cell cycle deregulation is a common feature of human cancers and, indeed, tumour cells frequently display unscheduled proliferation. The mammalian cell cycle is mainly controlled by CDKs, the activity of which is modulated by several activators (cyclins) and inhibitors. CDKs activity is commonly altered in cancer cells, and several human neoplasms present increased CDKs expression, indicating that these kinases are essential or play a relevant role for tumour growth and proliferation [5, 6].

One of the leader members of CDKs family is CDK2, which is centrally involved in cell cycle regulation together with CDK1, while the other CDKs appear to play an auxiliary role [18, 21]. Moreover, CDK2 has been reported to be essential for cell proliferation in several human tumours [4–6, 21] and emerging data have suggested that CDK1 or CDK2 can be considered as the most appropriate CDKs to target in combination with DNA-damaging agents [15].

Since CDK2 has not yet been analysed in detail in OS, the first part of our study focused on its evaluation in this neoplasm. We demonstrated that human OS clinical samples and cell lines present an inherent increased CDK2 expression compared to several human normal tissues, indicating that this kinase is relevant for OS biology. Moreover, patients with higher levels of CDK2 expression presented a trend toward a worse outcome suggesting that it may also impact on OS prognosis, despite this evidence needs further confirmation.

Based on this body of information, we proceeded with the evaluation of the biologic relevance of CDK2 for OS cell growth. Silencing experiment results clearly showed that CDK2 depletion reduced the proliferation of OS cells, indicating that negatively interfering with this kinase may inhibit OS cell growth. This evidence is in agreement with findings reported for other tumours, which demonstrated that downregulation or inhibition of CDK2 prevents cancer cells proliferation [4–6, 10, 37–39].

We therefore tested the activity of roscovitine, a CDKs inhibitor with high affinity for CDK2, on a panel of drug-sensitive and -resistant human OS cell lines. In the drug-sensitive cell lines, roscovitine proved to be markedly active, with IC50 values in a low micromolar range, as also described in other tumour cell lines [5, 6, 40]. Interestingly, roscovitine showed a comparable or even higher efficacy also in variants resistant to DX, CDDP or MTX indicating that its activity was not influenced by the drug resistant mechanisms developed by these cells. These findings indicated that roscovitine could be considered for treatment schedules in combination with conventional OS chemotherapeutic drugs.

Combined treatments demonstrated that roscovitine positively interacted with DX or CDDP, when it was sequentially administered after these two anticancer agents. This positive interaction is in agreement with the reported evidence that CDKs play an important role in DNA-damage-induced checkpoint control and repair, and that their inhibition increases sensitivity to DNA damaging agents [15].

On the other hand, association of roscovitine with CDDP mainly produced negative interactions. This findings can be explained by the fact that, in drug association treatments, the effectiveness of CDDP may be reduced by the cell cycle perturbations induced by roscovitine, as described in other experimental systems [15].

Association with MTX proved to be almost invariably antagonistic, most probably because of the cytostatic effects that roscovitine produced in OS cell lines, which negatively interfered with the activity of this S phase-specific drug. However, sequential treatment of MTX followed by roscovitine mainly resulted in additive interactions.

In our experimental models, roscovitine was also able to induce apoptosis, but only in Saos-2 and not in U-2OS cell lines. These results are concordant with what has been reported in the literature for OS, which demonstrated roscovitine-induced PARP-1 cleavage in Saos-2 (P53-negative) cells [41], while did not detect apoptosis in U-2OS (P53-positive) cells treated with roscovitine and/or DX [42]. These data indicated that in OS cells roscovitine-induced apoptosis may be mediated by P53, but this evidence needs to be further confirmed. In fact, the involvement of P53 in the induction of apoptosis by roscovitine in human tumour cells is still controversial, since this drug has been reported to induce cell death in several cell lines independently of P53 *status* [6]. For example, Mohapatra *et al* demonstrated that roscovitine preferentially induced apoptosis in prostate cancer and melanoma cells expressing wild-type P53 [43, 44], whereas in leukemia cells roscovitine-induced apoptosis resulted to be independent from the P53 *status* [45, 46].

Catalytic activities of CDKs also play a critical role in drug-induced DNA damage response [15, 17, 18, 36]. This fact has to be taken into consideration because we recently demonstrated that overexpression at diagnosis of the DNA repair-related factor excision repair cross-complementation group 1 (ERCC1) negatively correlated with outcome in patients with conventional OS [20], indicating that DNA repair activity may significantly impact on OS treatment response and clinical course.

Since three out of the four drugs that are most frequently used for OS chemotherapy (namely DX, CDDP and ifosfamide) produced DNA damages, we investigated whether the roscovitine-induced CDK2-inhibition could also negatively interfere with the DNA repair activity in OS cells, therefore increasing their sensitivity to DNA damaging drugs. Our results provided evidence that roscovitine treatment delays the normal activation of DNA repair systems, leading to an accumlation of DNA double strand breaks and, consequently, to an increase of DX and CDDP cytotoxicity. This evidence indicated that inhibition of CDK2 and other roscovitine-targeted CDKs can successfully synergise with DNA-damaging agents used in OS treatment and could therefore be considered for clinical purposes.

In conclusion, our results indicated that combined treatment with conventional OS chemotherapeutic drugs and roscovitine may represent a new candidate intervention approach, which may be taken into consideration to improve conventional chemotherapy effectiveness. This indication is in agreement with other studies, which proved that roscovitine can increase conventional chemotherapy efficacy in sarcomas [42] and other tumours [5, 6, 47–49]. This perspective has a particular clinical relevance for OS patients who are unresponsive to standard treatments, because of inherent or acquired drug resistance, and even more for relapsed patients, for whom no effective treatment strategies are available yet [1–3].

## Supporting Information

S1 Fig **Original films of CDK2 and Actin protein detection by western blot after siRNA transfection** in U-2OS (A) and Saos-2 (B) human osteosarcoma cell lines. CDK2 and actin protein levels were assessed at different time points after the end of siRNA treatment (from 24- to 72 h for U-2OS; from 48- to 120 h for Saos-2). Legend: CTR, control, not-treated cells; SCR, cells transfected with scrambled siRNA; CDK2, cells transfected with anti-CDK2 siRNA; AURKA, cells transfected with anti-Aurora Kinase siRNA; h, hours after siRNA transfection. In Saos-2 films, samples 1, 4, 7, and 10 are control, not-treated cells; samples 2, 5, 8, and 11 are cells transfected with scrambled siRNA; samples 3, 6, 9, and 12 are cells transfected with anti-CDK2 siRNA.
(JPG)

S2 Fig Relative CDK2 expression level in the drug-resistant variants in comparison to their parental cell lines, assessed by qRT-PCR and western blot. Data refer to one representative experiment. (JPG)

S3 Fig Isobolograms illustrating the interaction between roscovitine with conventional chemotherapeutic drugs, which were used to calculate the combination indexes listed in Table 2. A) Drug association experiments. B) Drug sequence experiments. Graphs refer to one representative experiment.
(PDF)

S4 Fig **Original western blot films for the analyisis of cleaved caspase 3 and relative actin on U-2OS/DX580 and U-2OS/CDDP4μg cell lines (A) and of PARP-1 and relative actin on Saos-2 and Saos-2/DX580 (B).** Legend: CTR 24h, control, not-treated cells harvested after 24h from seeding; CTR 72h, control, not-treated cells harvested after 72h from seeding; DX 24h, CDDP 24h, ROS 48h, cells treated with their respective IC50 dosage of doxorubicin (DX), cisplatin (CDDP) or roscovitine (ROS) harvested after 24h or 48h of treatment; SEQ, cells sequentially treated with DX or CDDP for 24h followed by roscovitine ROS for 48h. Positive controls (last lane) are represented by U-2OS and Saos-2 cell lines treated, respectively, with 5 μg/ml or 10 μg/ml CDDP for 48 h.
(TIF)

S1 Table Effects on cell cycle of doxorubicin (DX), cisplatin (CDDP) and roscovitine (ROS). (DOCX)
