# Supplementary figures and images for: Targeting CDKs with Roscovitine Increases Sensitivity to DNA Damaging Drugs of Human Osteosarcoma Cells
Source: PLoS One. 2016 Nov 29;11(11):e0166233. doi: 10.1371/journal.pone.0166233 (PMC5127503; doi:10.1371/journal.pone.0166233)

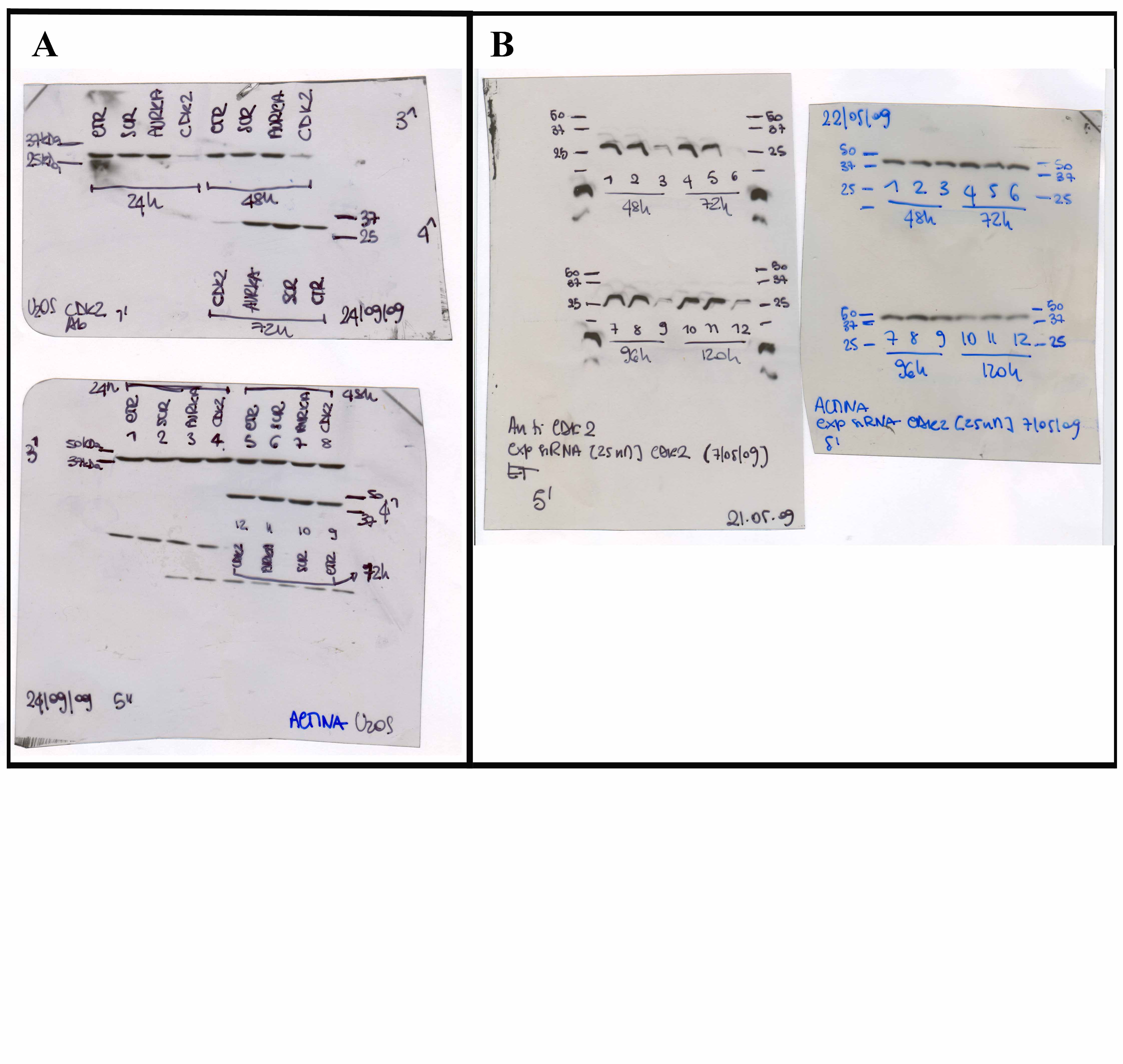

Supplement: S1 Fig — Original films of CDK2 and Actin protein detection by western blot after siRNA transfection in U-2OS (A) and Saos-2 (B) human osteosarcoma cell lines. CDK2 and actin protein levels were assessed at different time points after the end of siRNA treatment (from 24- to 72 h for U-2OS; from 48- to 120 h for Saos-2). Legend: CTR, control, not-treated cells; SCR, cells transfected with scrambled siRNA; CDK2, cells transfected with anti-CDK2 siRNA; AURKA, cells transfected with anti-Aurora Kinase siRNA; h, hours after siRNA transfection. In Saos-2 films, samples 1, 4, 7, and 10 are control, not-treated cells; samples 2, 5, 8, and 11 are cells transfected with scrambled siRNA; samples 3, 6, 9, and 12 are cells transfected with anti-CDK2 siRNA. (JPG) [file pone.0166233.s001.jpg]

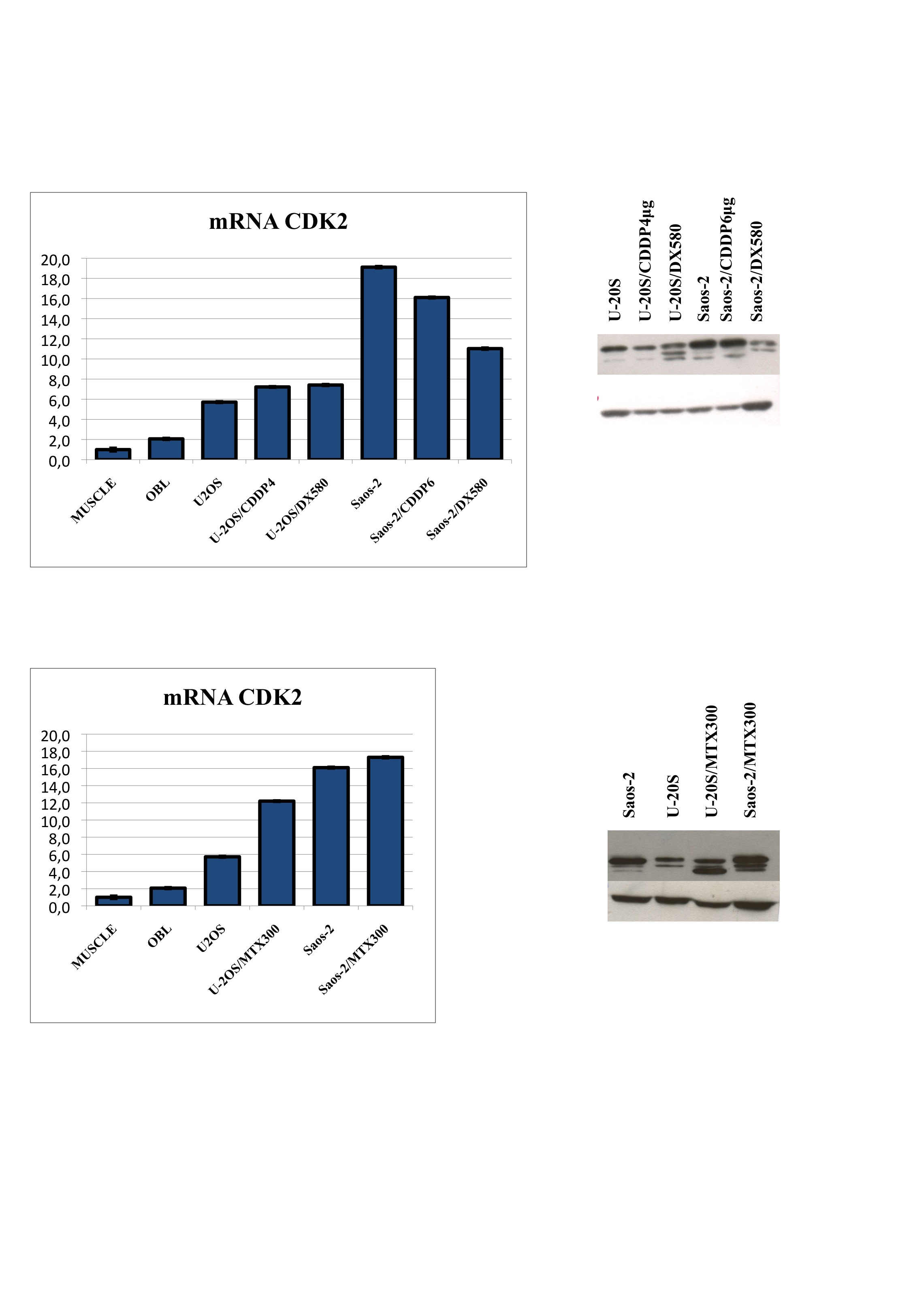

Supplement: S2 Fig — (JPG) [file pone.0166233.s002.jpg]

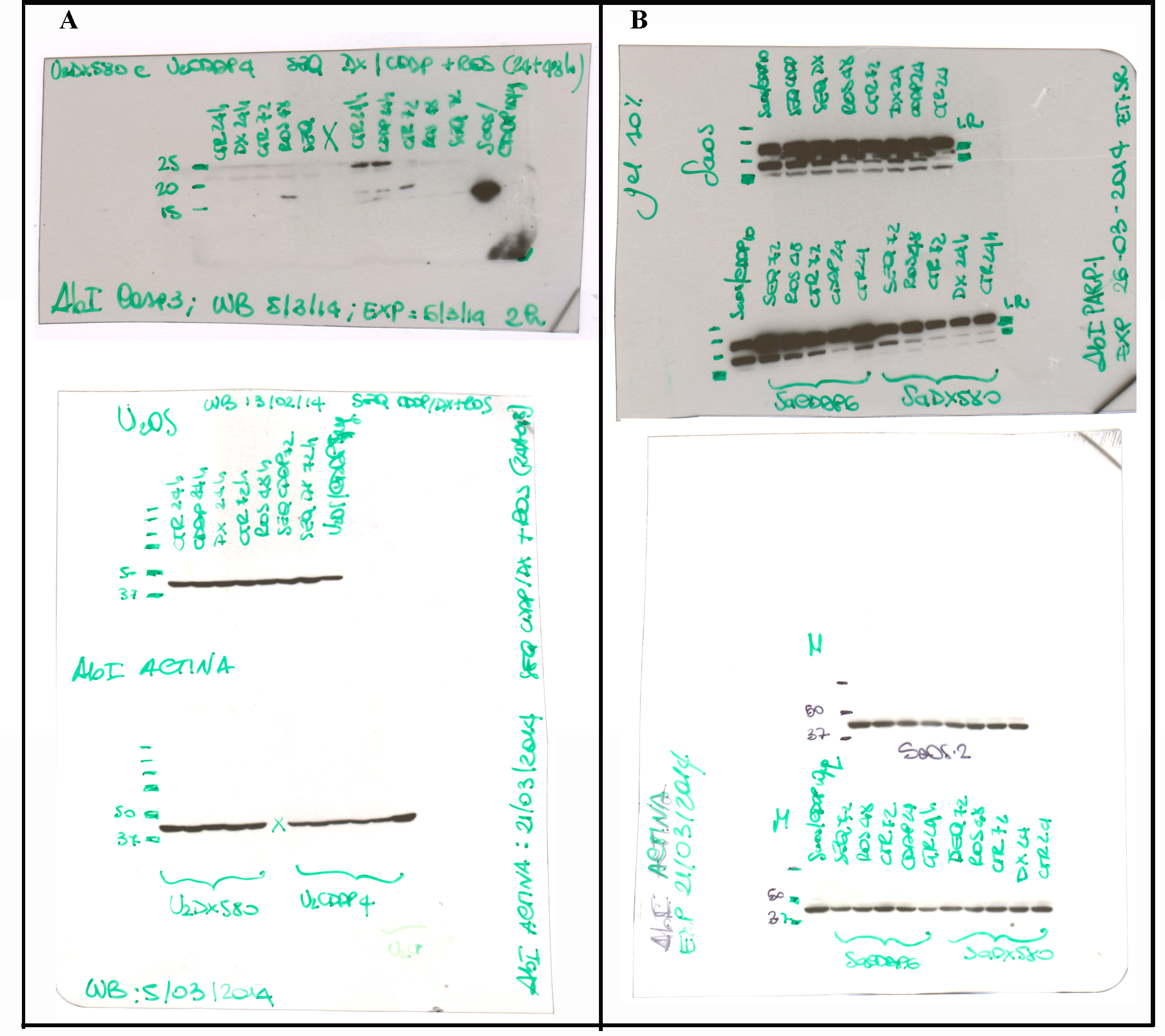

Supplement: S4 Fig — Original western blot films for the analyisis of cleaved caspase 3 and relative actin on U-2OS/DX580 and U-2OS/CDDP4μg cell lines (A) and of PARP-1 and relative actin on Saos-2 and Saos-2/DX580 (B). Legend: CTR 24h, control, not-treated cells harvested after 24h from seeding; CTR 72h, control, not-treated cells harvested after 72h from seeding; DX 24h, CDDP 24h, ROS 48h, cells treated with their respective IC50 dosage of doxorubicin (DX), cisplatin (CDDP) or roscovitine (ROS) harvested after 24h or 48h of treatment; SEQ, cells sequentially treated with DX or CDDP for 24h followed by roscovitine ROS for 48h. Positive controls (last lane) are represented by U-2OS and Saos-2 cell lines treated, respectively, with 5 μg/ml or 10 μg/ml CDDP for 48 h. (TIF) [file pone.0166233.s004.tif]
