# Supplementary material for: Targeting CDKs with Roscovitine Increases Sensitivity to DNA Damaging Drugs of Human Osteosarcoma Cells
Source: PLoS One. 2016 Nov 29;11(11):e0166233. doi: 10.1371/journal.pone.0166233 (PMC5127503; doi:10.1371/journal.pone.0166233)

# A) DRUG ASSOCIATION

## DX+Roscovitine

U-2OS

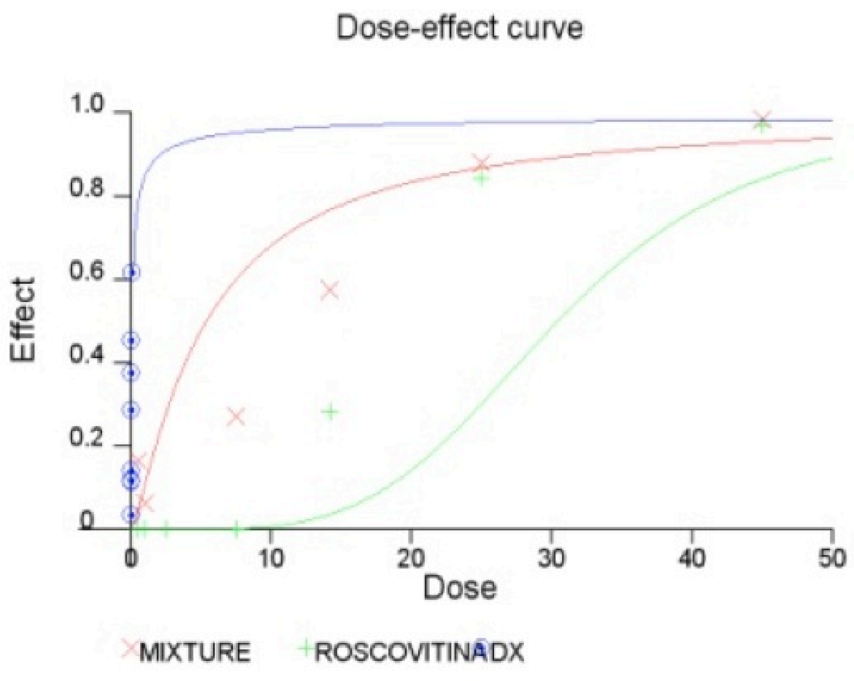


Saos-2

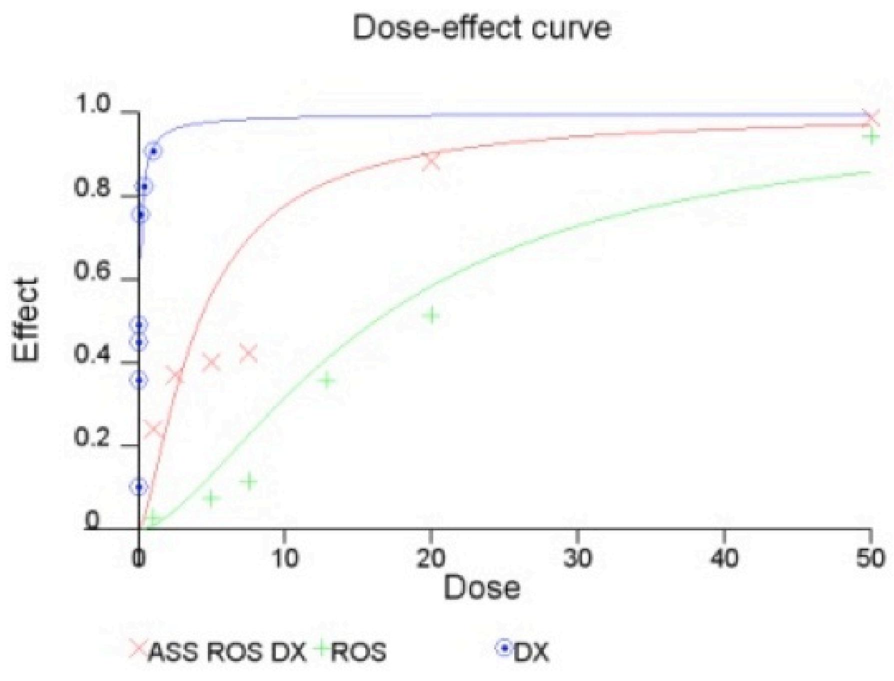


U-2OS/DX580

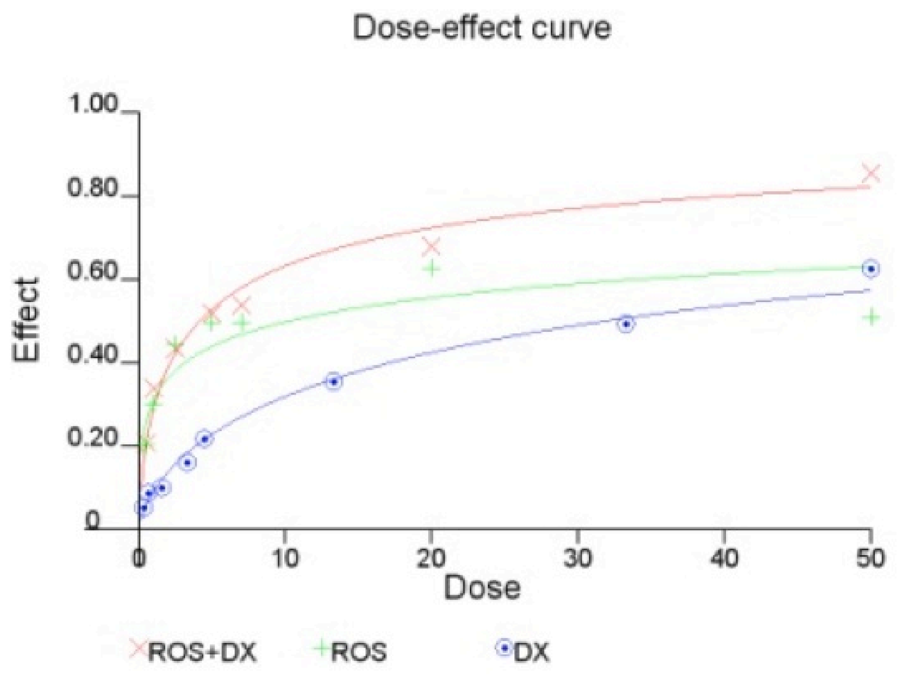


Saos-2/DX580

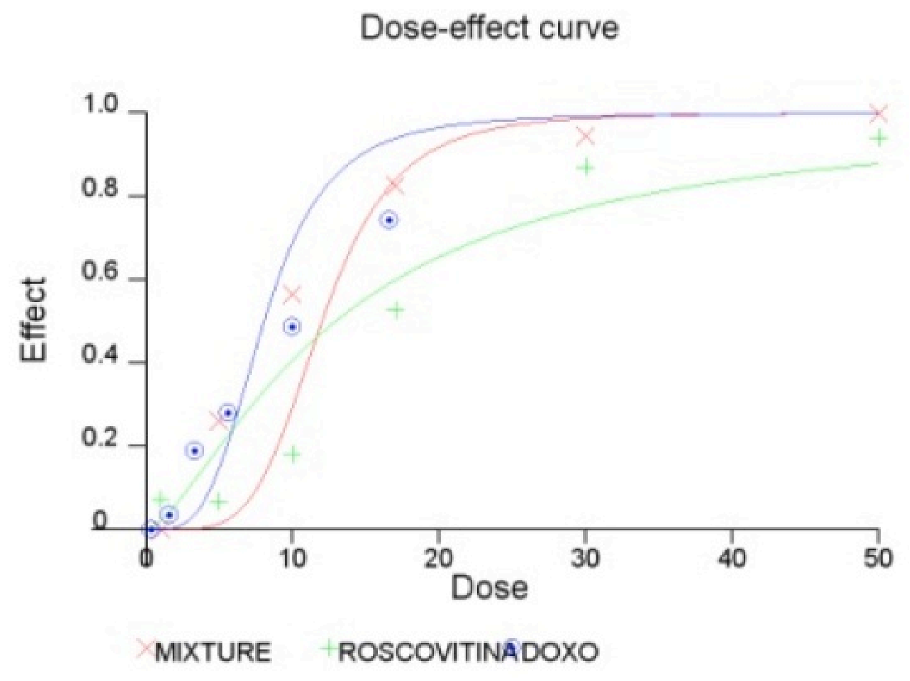


## MTX+Roscovitine

U-2OS

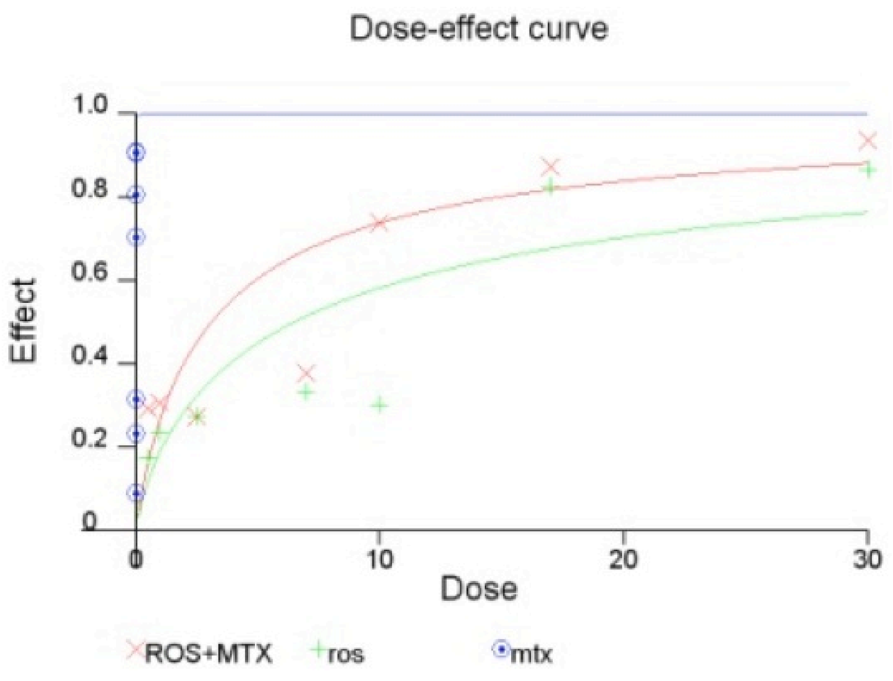


Saos-2

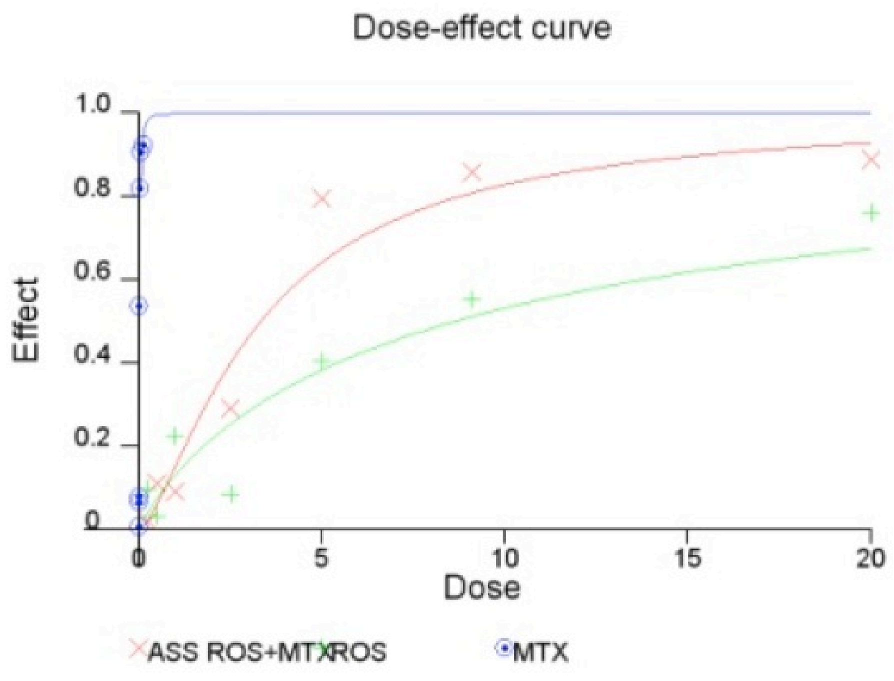


U-2OS/MTX300

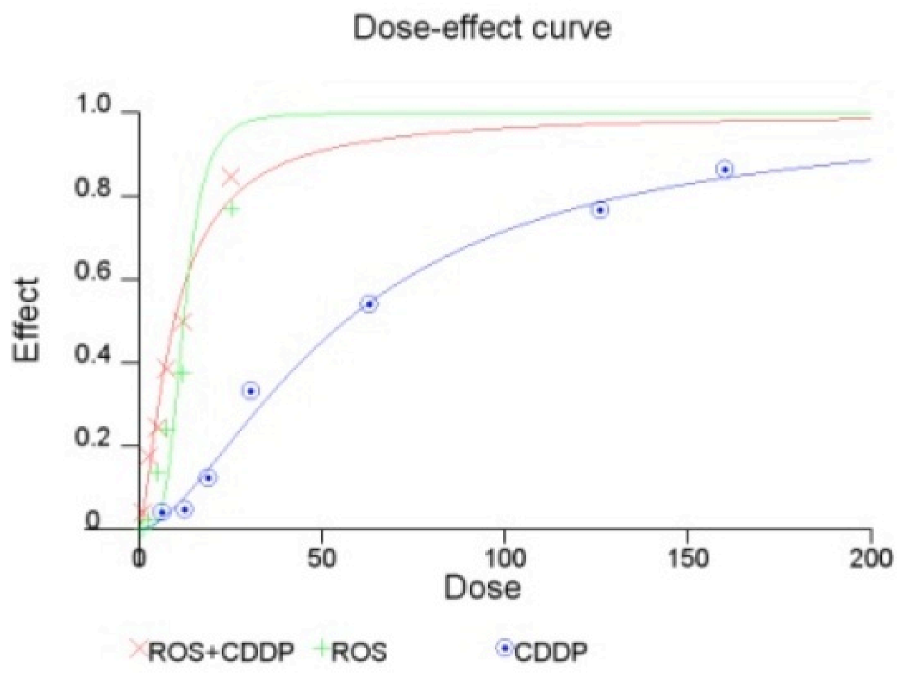


Saos-2/MTX300

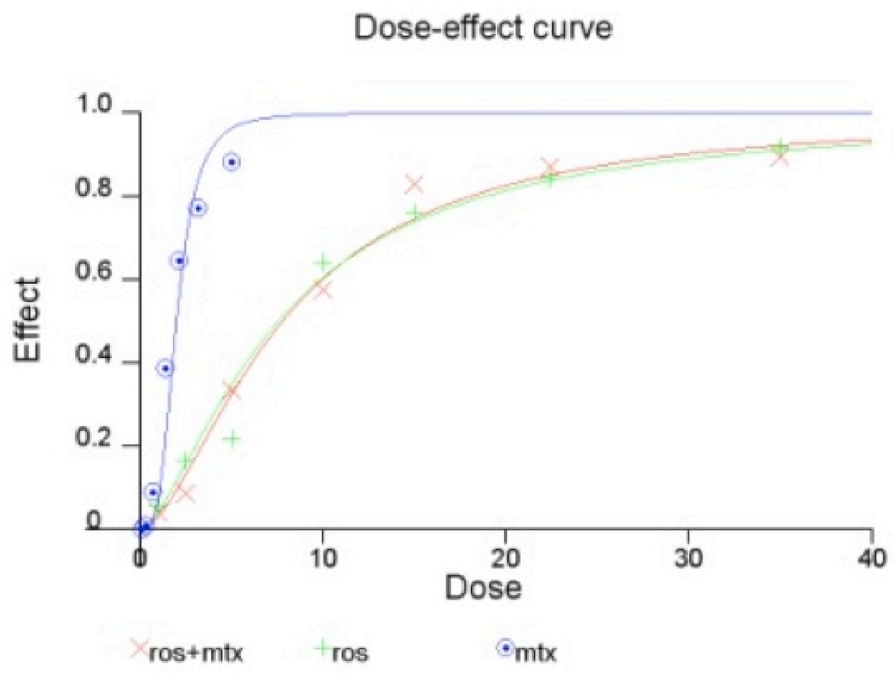


## CDDP+Roscovitine

U-2OS

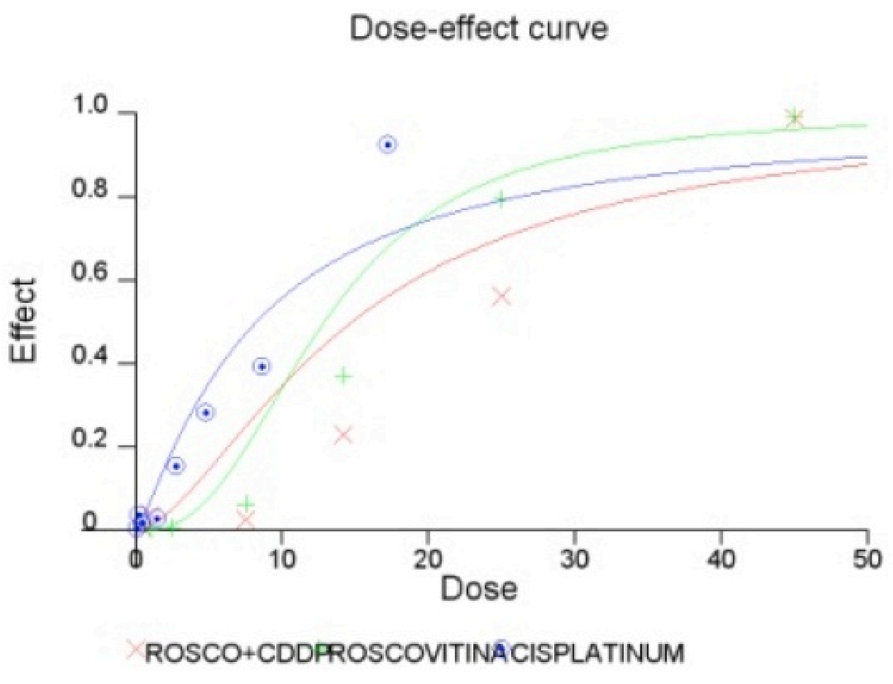


Saos-2

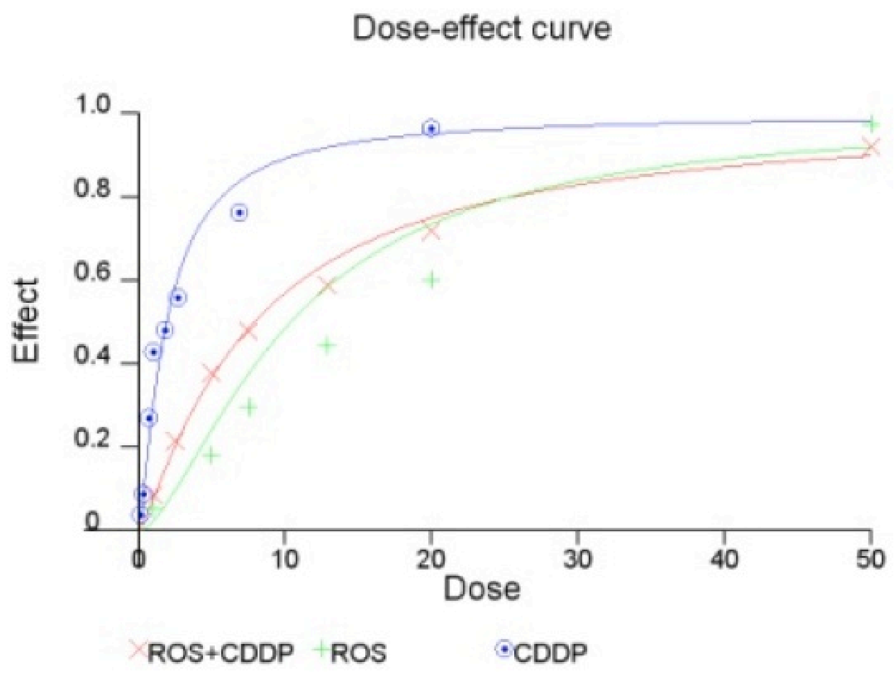


U-2OS/CDDP4µg

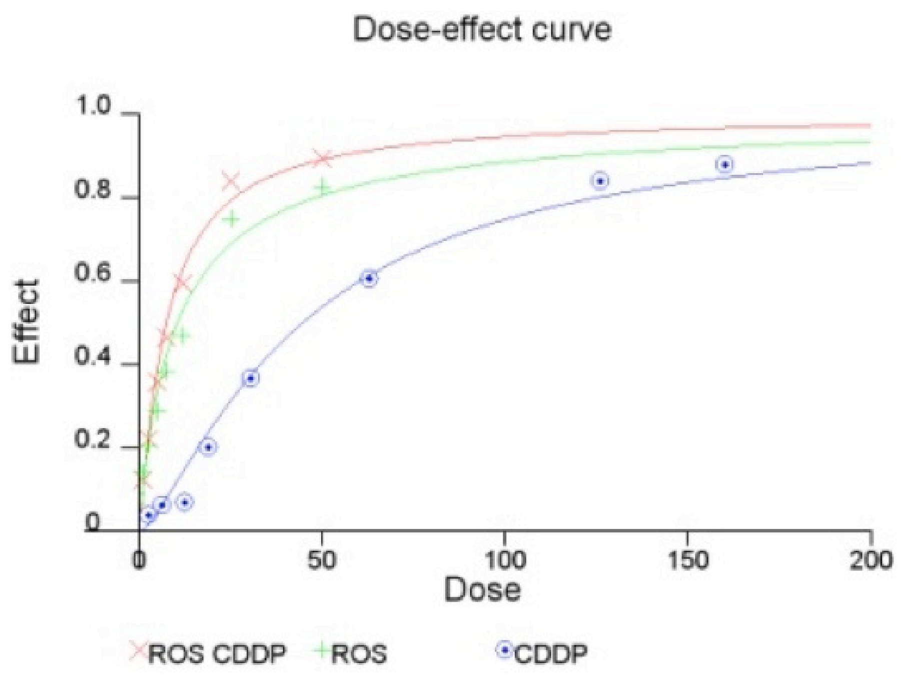


Saos-2/CDDP6µg

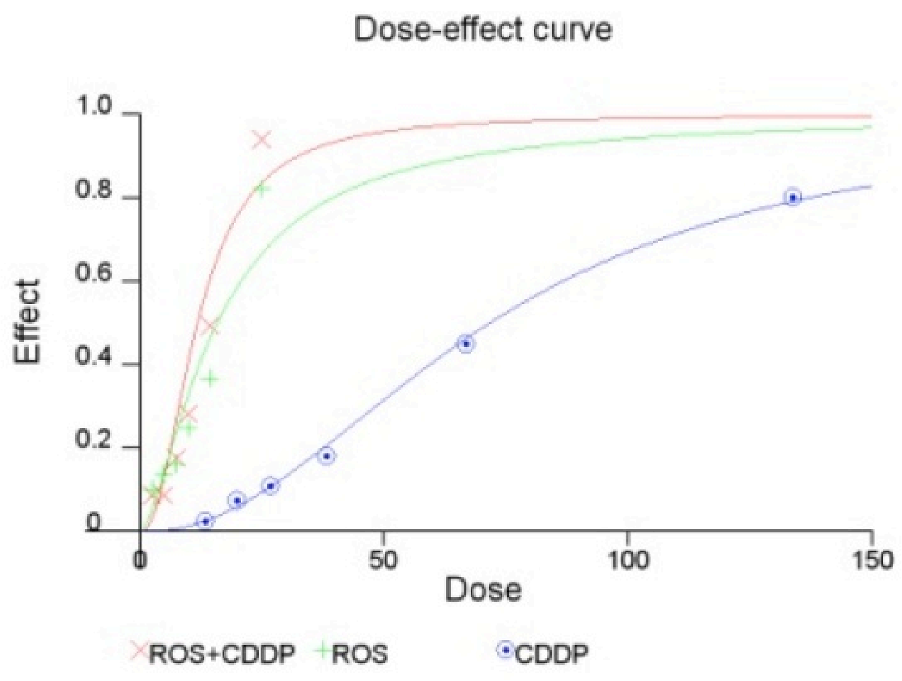

# B) DRUG SEQUENCE

## Roscovitine+DX

### U-2OS

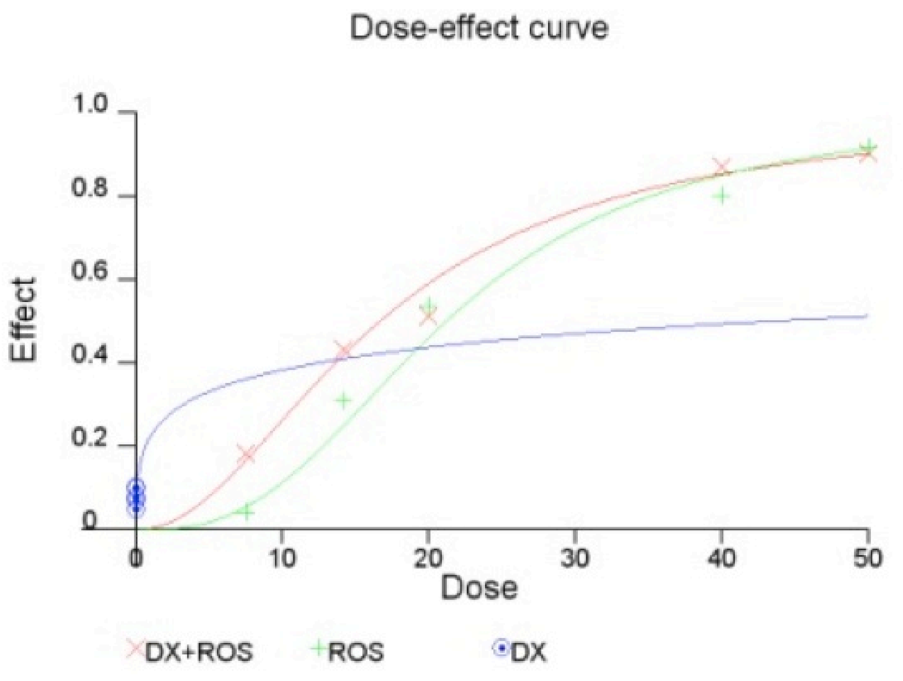


### Saos-2

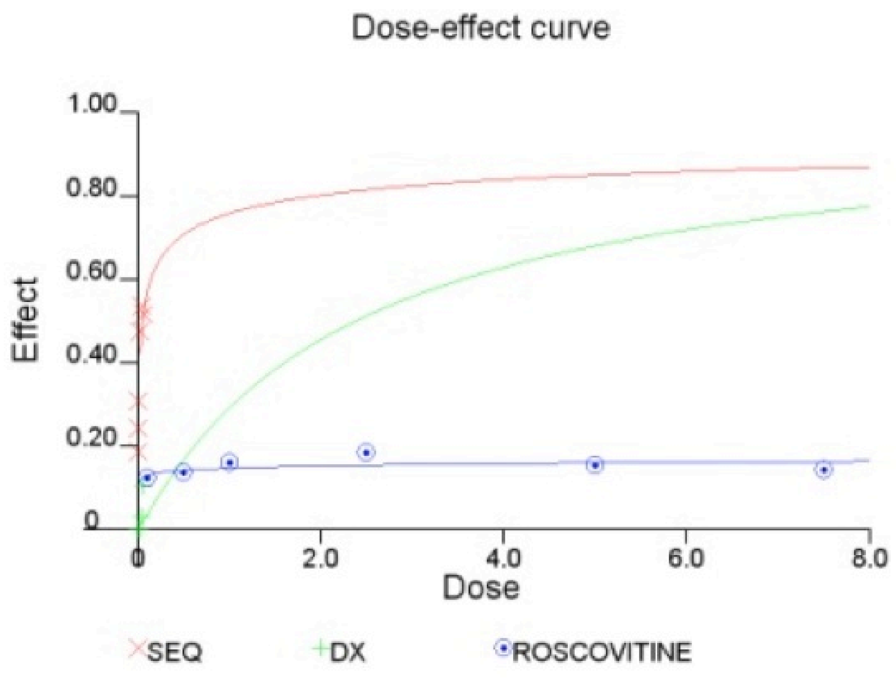


### U-2OS/DX580

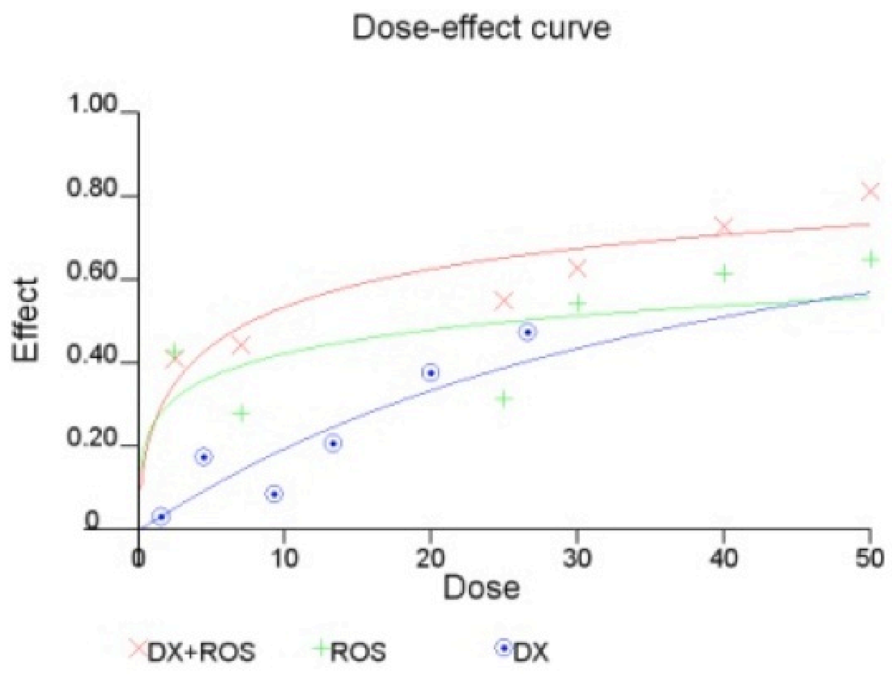


### Saos-2/DX580

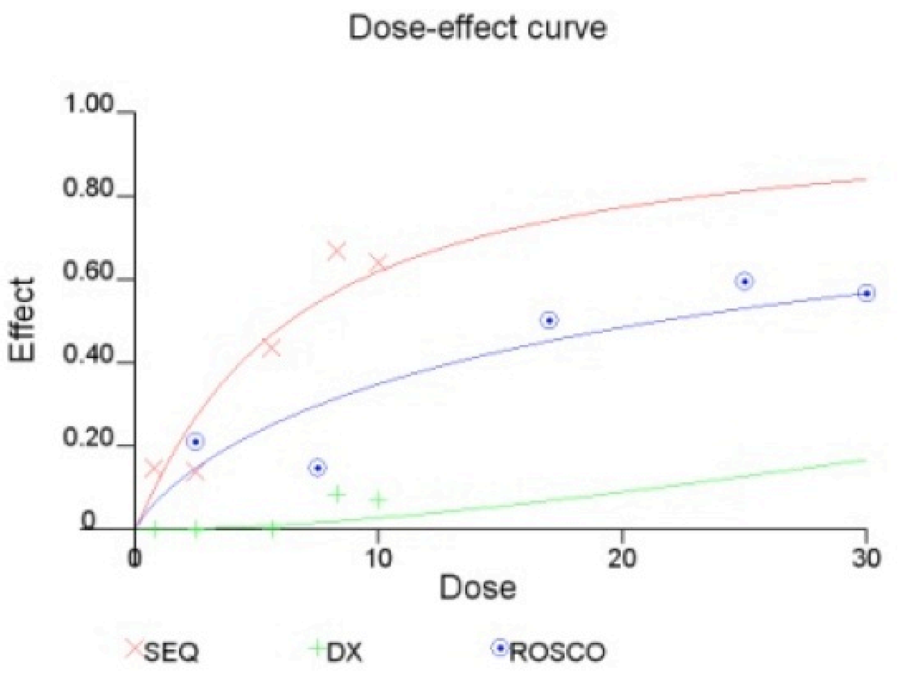


## Roscovitine+MTX

### U-2OS

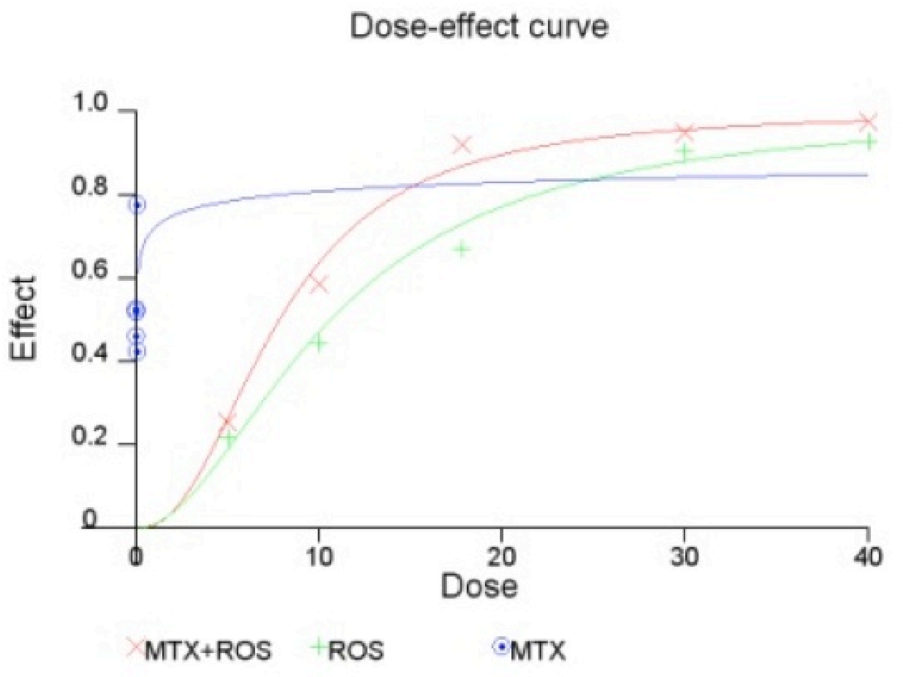


### Saos-2

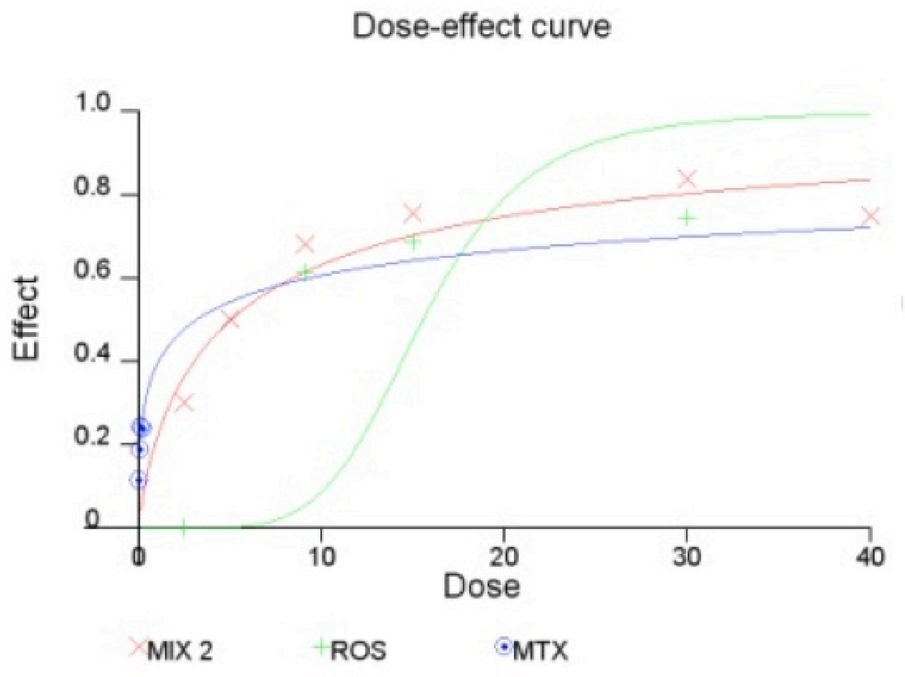


### U-2OS/MTX300

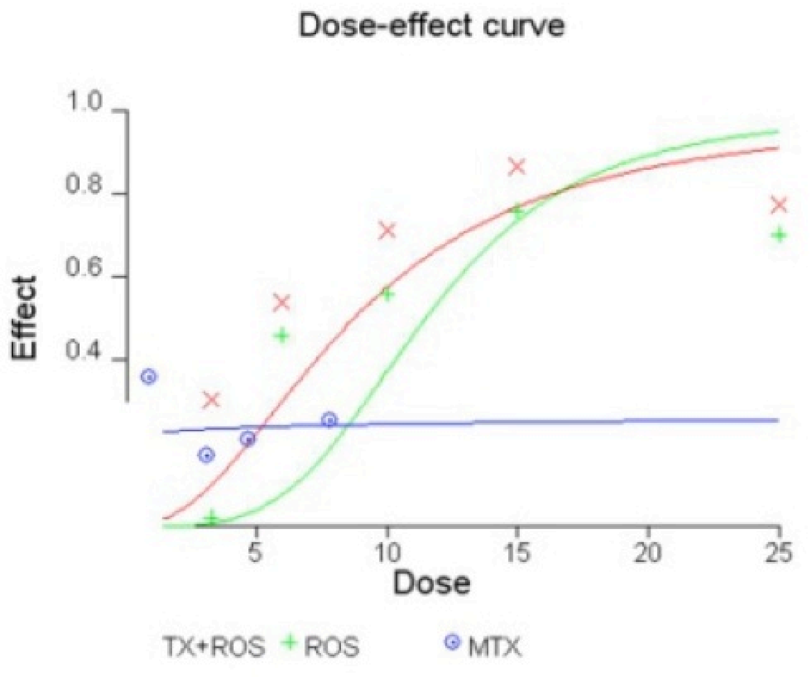


### Saos-2/MTX300

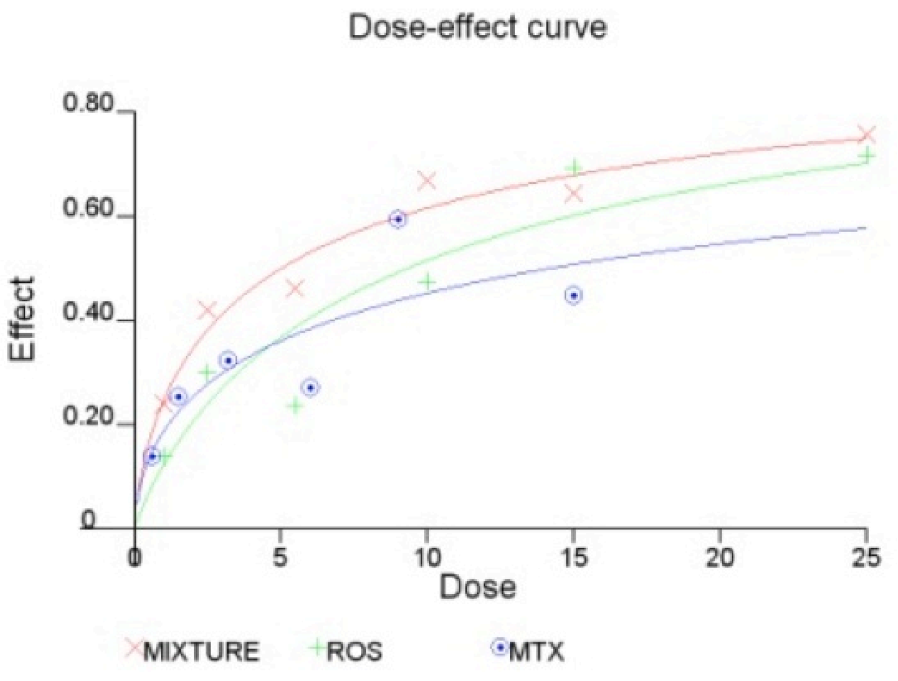


## Roscovitine+CDDP

### U-2OS

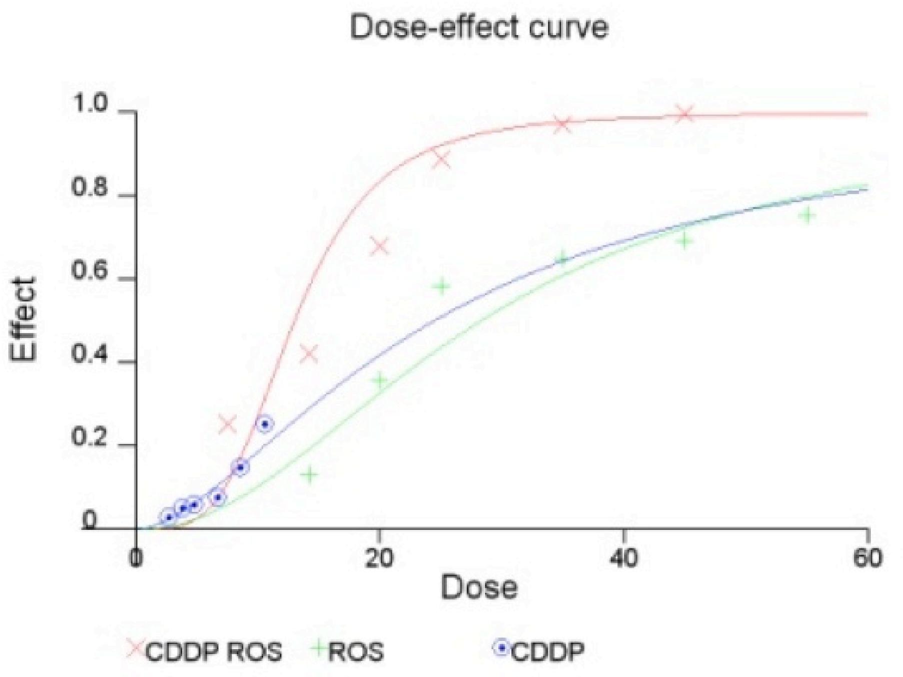


### Saos-2

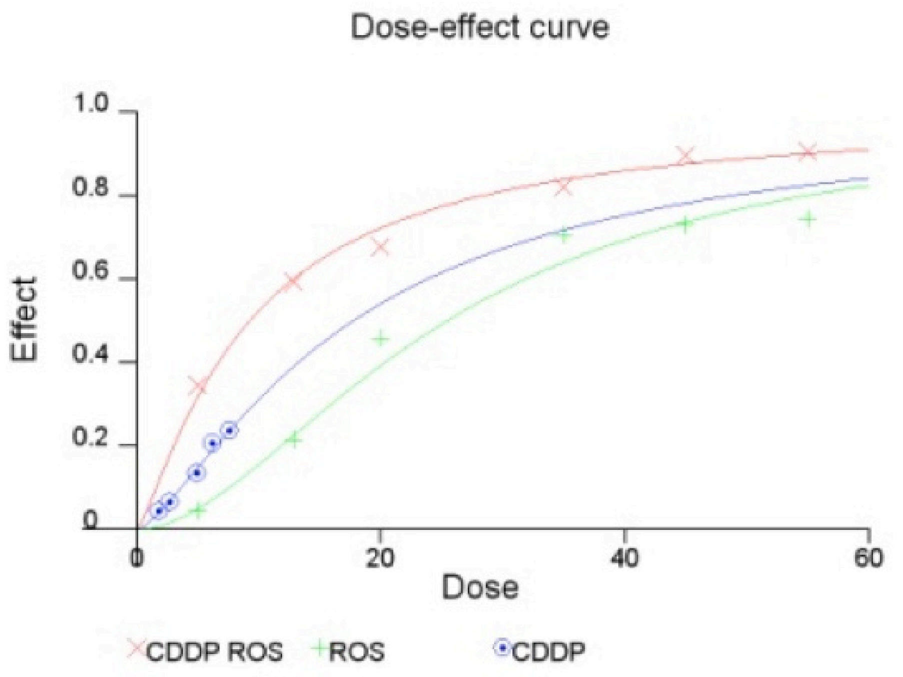


### U-2OS/CDDP4µg

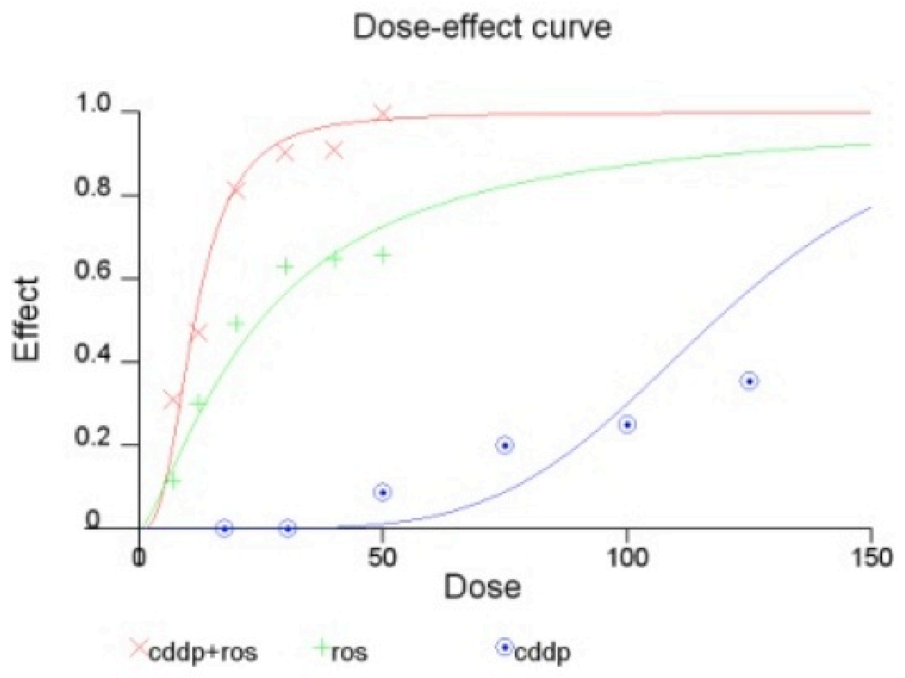


### Saos-2/CDDP6µg

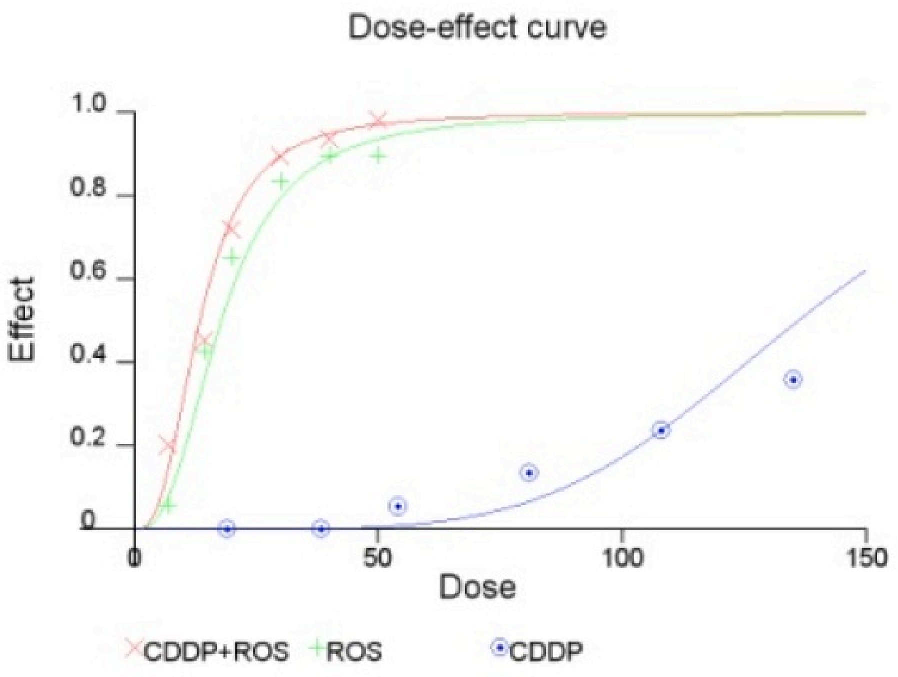

Supplement: S3 Fig — A) Drug association experiments. B) Drug sequence experiments. Graphs refer to one representative experiment. (PDF) [file pone.0166233.s003.pdf]
