# Supplementary material for: Targeting CDKs with Roscovitine Increases Sensitivity to DNA Damaging Drugs of Human Osteosarcoma Cells
Source: PLoS One. 2016 Nov 29;11(11):e0166233. doi: 10.1371/journal.pone.0166233 (PMC5127503; doi:10.1371/journal.pone.0166233)
Supplement: S1 Table — (DOCX) [file pone.0166233.s005.docx]

**S1 Table. Effects on cell cycle of doxorubicin (DX), cisplatin (CDDP) and roscovitine (ROS)**.

| **U-2OS** | | | | | | |
| --- | --- | --- | --- | --- | --- | --- |
| **%GATED** | CTR | DX IC50 | CDDP IC50 | ROSCO IC50 | DX IC50 >ROS IC50 | CDDP IC50>ROS IC50 |
| S | 55.56 | 24.65 | 76.00 | 45.50 | 5.98 | 34.46 |
| G0/G1 | 27.80 | 4.52 | 7.17 | 38.38 | 0.32 | 0.82 |
| G2/M | 16.06 | 68.65 | 16.44 | 15.39 | 8.47 | 7.41 |
| Apoptosis | 0.31 | 0.96 | 0.27 | 0.31 | 2.47 | 1.31 |
| Hyperploid | 0.25 | 1.20 | 0.24 | 0.42 | 82.97 | 38.65 |

| **Saos-2** | | | | | | |
| --- | --- | --- | --- | --- | --- | --- |
| **%GATED** | CTR | DX IC50 | CDDP IC50 | ROSCO IC50 | DX IC50 >ROS IC50 | CDDP IC50>ROS IC50 |
| S | 32.00 | 59.40 | 66.87 | 33.70 | 12.53 | 52.84 |
| G0/G1 | 55.52 | 8.10 | 20.16 | 34.70 | 2.05 | 3.38 |
| G2/M | 11.27 | 25.21 | 10.27 | 27.39 | 51.34 | 34.06 |
| Apoptosis | 0.97 | 6.59 | 2.52 | 3.25 | 5.21 | 2.13 |
| Hyperploid | 0.55 | 0.86 | 0.24 | 1.25 | 28.87 | 7.77 |

| **U-2OS/DX580** | | | | | | |
| --- | --- | --- | --- | --- | --- | --- |
| **%GATED** | CTR | DX IC50 | CDDP IC50 | ROSCO IC50 | DX IC50 >ROS IC50 | CDDP IC50>ROS IC50 |
| S | 48.67 | 30.53 |  | 24.87 | 19.27 |  |
| G0/G1 | 34.44 | 36.73 |  | 51.95 | 40.92 |  |
| G2/M | 15.29 | 31.61 |  | 21.14 | 37.10 |  |
| Apoptosis | 1.23 | 0.40 |  | 1.12 | 1.05 |  |
| Hyperploid | 0.74 | 0.72 |  | 0.82 | 1.52 |  |

| **Saos-2/DX580** | | | | | | |
| --- | --- | --- | --- | --- | --- | --- |
| **%GATED** | CTR | DX IC50 | CDDP IC50 | ROSCO IC50 | DX IC50 >ROS IC50 | CDDP IC50>ROS IC50 |
| S | 34.69 | 41.72 |  | 46.54 | 43.35 |  |
| G0/G1 | 47.85 | 36.00 |  | 23.88 | 16.08 |  |
| G2/M | 16.04 | 20.89 |  | 20.81 | 28.83 |  |
| Apoptosis | 0.65 | 0.73 |  | 2.04 | 2.62 |  |
| Hyperploid | 0.76 | 0.53 |  | 6.83 | 9.35 |  |

| **U-2OS/CDDP4μg** | | | | | | |
| --- | --- | --- | --- | --- | --- | --- |
| **%GATED** | CTR | DX IC50 | CDDP IC50 | ROSCO IC50 | DX IC50 >ROS IC50 | CDDP IC50>ROS IC50 |
| S | 29.49 |  | 60.81 | 28.45 |  | 29.60 |
| G0/G1 | 41.83 |  | 19.80 | 52.69 |  | 13.67 |
| G2/M | 25.06 |  | 15.95 | 14.50 |  | 42.52 |
| Apoptosis | 2.07 |  | 2.88 | 3.80 |  | 2.50 |
| Hyperploid | 1.71 |  | 0.62 | 0.39 |  | 11.77 |

| **Saos-2/CDDP6μg** | | | | | | |
| --- | --- | --- | --- | --- | --- | --- |
| **%GATED** | CTR | DX IC50 | CDDP IC50 | ROSCO IC50 | DX IC50 >ROS IC50 | CDDP IC50>ROS IC50 |
| S | 33.14 |  | 63.40 | 41.65 |  | 52.89 |
| G0/G1 | 54.66 |  | 28.18 | 28.17 |  | 1.18 |
| G2/M | 8.92 |  | 5.64 | 18.40 |  | 6.35 |
| Apoptosis | 2.93 |  | 2.71 | 10.94 |  | 12.60 |
| Hyperploid | 0.34 |  | 0.18 | 0.60 |  | 27.47 |

Effects on cell cycle of doxorubicin (DX), cisplatin (CDDP) and roscovitine (ROS), used either alone or in combination, in the human osteosarcoma cell lines U-2OS and Saos-2 and their variants resistant to DX or CDDP. Cell cycle phase distribution was determined by flow cytometry after bromodeoxyuridine (BrdU) incorporation and propidium iodide counterstaining. Legend: CTR, control cells cultured in drug-free medium; DX, CDDP, ROS, cells treated with the IC50 dose of each drug for 24 h (DX and CDDP) or 48 h (ROS); DX>ROS and CDDP>ROS, cells sequentially treated with the IC50 dose of DX or CDDP for 24 h followed by treatment with the IC50 dose of ROS for 48 h.
